# Improving the measurement of alexithymia in autistic adults: a psychometric investigation of the 20-item Toronto Alexithymia Scale and generation of a general alexithymia factor score using item response theory

**DOI:** 10.1186/s13229-021-00463-5

**Published:** 2021-08-10

**Authors:** Zachary J. Williams, Katherine O. Gotham

**Affiliations:** 1 Medical Scientist Training Program, Vanderbilt University School of Medicine, 1215 21st Avenue South, Medical Center East, Room 8310, Nashville, TN 37232 USA; 2 Department of Hearing and Speech Sciences, Vanderbilt University Medical Center, Nashville, TN USA; 3 Vanderbilt Brain Institute, Vanderbilt University, Nashville, TN USA; 4 Frist Center for Autism and Innovation, Vanderbilt University, Nashville, TN USA; 5 Department of Psychology, Rowan University, Glassboro, NJ USA

**Keywords:** Autism, Alexithymia, Bayesian statistics, Differential item functioning, Emotion, Item response theory, Factor analysis, Measurement, Psychometric, Reliability, Validity

## Abstract

**Background:**

Alexithymia, a personality trait characterized by difficulties interpreting emotional states, is commonly elevated in autistic adults, and a growing body of literature suggests that this trait underlies several cognitive and emotional differences previously attributed to autism. Although questionnaires such as the 20-item Toronto Alexithymia Scale (TAS-20) are frequently used to measure alexithymia in the autistic population, few studies have investigated the psychometric properties of these questionnaires in autistic adults, including whether differential item functioning (I-DIF) exists between autistic and general population adults.

**Methods:**

This study is a revised version of a previous article that was retracted due to copyright concerns (Williams and Gotham in Mol Autism 12:1–40). We conducted an in-depth psychometric analysis of the TAS-20 in a large sample of 743 cognitively able autistic adults recruited from the Simons Foundation SPARK participant pool and 721 general population controls enrolled in a large international psychological study. The factor structure of the TAS-20 was examined using confirmatory factor analysis, and item response theory was used to generate a subset of the items that were strong indicators of a “general alexithymia” factor. Correlations between alexithymia and other clinical outcomes were used to assess the nomological validity of the new alexithymia score in the SPARK sample.

**Results:**

The TAS-20 did not exhibit adequate model fit in either the autistic or general population samples. Empirically driven item reduction was undertaken, resulting in an 8-item general alexithymia factor score (GAFS-8, with “TAS” no longer referenced due to copyright) with sound psychometric properties and practically ignorable I-DIF between diagnostic groups. Correlational analyses indicated that GAFS-8 scores, as derived from the TAS-20, meaningfully predict autistic trait levels, repetitive behaviors, and depression symptoms, even after controlling for trait neuroticism. The GAFS-8 also presented no meaningful decrement in nomological validity over the full TAS-20 in autistic participants.

**Limitations:**

Limitations of the current study include a sample of autistic adults that was majority female, later diagnosed, and well educated; clinical and control groups drawn from different studies with variable measures; only 16 of the TAS-20 items being administered to the non-autistic sample; and an inability to test several other important psychometric characteristics of the GAFS-8, including sensitivity to change and I-DIF across multiple administrations.

**Conclusions:**

These results indicate the potential of the GAFS-8 to robustly measure alexithymia in both autistic and non-autistic adults. A free online score calculator has been created to facilitate the use of norm-referenced GAFS-8 latent trait scores in research applications (available at https://asdmeasures.shinyapps.io/alexithymia).

**Supplementary Information:**

The online version contains supplementary material available at 10.1186/s13229-021-00463-5.

## Background

Alexithymia is a subclinical construct characterized by difficulties in identifying and describing one’s own emotional state [2, 3]. Individuals scoring high on measures of alexithymia exhibit difficulties recognizing and labeling their internal emotional states, discriminating between different emotions of the same affective valence, and describing and communicating their emotional states to others. These individuals also tend to exhibit a reduction in imaginal processes and a stimulus-bound, externally oriented style of thinking (i.e., “concrete thinking”). Alexithymia is not itself considered a psychiatric diagnosis; rather, the condition can better be described as a dimensional personality trait that is expressed to varying degrees in the general population and associated with a host of medical, psychiatric, and psychosomatic conditions [3–15]. Although there is taxometric evidence to suggest that alexithymia is a dimensional rather than categorical construct [16–18], researchers frequently categorize a portion of individuals as having “high alexithymia” based on questionnaire scores above a certain threshold, with upward of 10% of the general population exceeding these thresholds [19–21]. Over the last five decades, a large body of research has emerged to suggest that alexithymia is a transdiagnostic predictor of important clinical outcomes, such as the presence of psychiatric and psychosomatic disorders, suicidal ideation and behavior, non-suicidal self-injury, risky drinking, and reduced response to various medical and psychotherapeutic treatments [22–27].

Alexithymia is a construct of particular interest in research on autism spectrum disorder (hereafter “autism”), a condition frequently associated with difficulties in processing, recognizing, communicating, and regulating emotions [28–33]. A recent meta-analysis of published studies identified large differences between autistic adolescents/adults and neurotypical controls on self-reported alexithymia as measured by variants of the Toronto Alexithymia Scale (TAS [3, 34, 35]), with an estimated 49.93% of autistic individuals exceeding cutoffs for “high alexithymia” on the 20-item TAS (TAS-20), compared to only 4.89% of controls [4]. Alexithymia has also been suggested to be part of the “Broader Autism Phenotype” [36–38], the cluster of personality characteristics observed in parents of autistic children and other individuals with high-levels of subclinical autistic traits [39]. Along with verbal IQ, self-reported alexithymia is one of the stronger predictors of task-based emotion-processing ability in the autistic population [30], and a number of studies measuring both alexithymia and core autism symptoms have concluded that alexithymia accounts for some or all of the emotion-processing differences associated with the categorical diagnosis of autism, such as impaired facial emotion recognition and differences in empathetic responses [40–53]. Within the autistic population, alexithymia is also a meaningful predictor of the severity of co-occurring mental health conditions, showing relationships with symptoms of depression, general anxiety, social anxiety, non-suicidal self-injury, and suicidality [54–61].

Despite the impressive body of literature on alexithymia in autistic individuals and its relationships with other constructs, there has been surprisingly little investigation into the measurement properties of alexithymia measures in the autistic population [62]. One small study by Berthoz and Hill [63] addressed the validity of two common alexithymia scales (the TAS-20 and Bermond–Vorst Alexithymia Questionnaire-Form B [BVAQ-B] [64]) in a sample of 27 autistic adults and 35 neurotypical controls. In this small sample, the investigators found that autistic adults adequately comprehended the content of the alexithymia questionnaires, also noting high correlations between the two measures in both diagnostic groups. A subset of the sample also completed the same forms 4–12 months later, and test–retest reliability coefficients for both the TAS-20 and BVAQ-B in autistic adults were deemed adequate (test–retest Pearson *r* = 0.92 and 0.81 for the TAS-20 and BVAQ-B total scores, respectively, with all subscale *r*s > 0.62). The internal consistency of the TAS-20 and its three subscales has also been reported in a sample of 27 autistic adults by Samson et al. [65], who reported adequate reliability for the TAS-20 total score (*α* = 0.84), “difficulty identifying feelings” (DIF) subscale (*α* = 0.76), and “difficulty describing feelings” (DDF) subscale (*α* = 0.81) subscales, but subpar reliability for the TAS-20 “externally oriented thinking” (EOT) subscale (*α* = 0.65). Additional studies have also replicated the high correlations between TAS-20 and BVAQ scores in autistic adults [43] and demonstrated the TAS-20 total score and combined DIF/DDF subscales to be reliable in samples of cognitively able autistic adolescents [52, 58]. Nevertheless, we are unaware of any study to date systematically investigating the psychometric properties of the TAS-20 or any other alexithymia measure in autistic individuals using large-sample latent variable modeling techniques.

Given the prominence of the TAS-20 as the primary alexithymia measure employed in autism literature [4, 30, 62], the remainder of this paper will focus specifically on this scale. Although the TAS-20 is extensively used in research on alexithymia in a number of clinical and non-clinical populations [3], a number of psychometric concerns have been raised about the measure’s factor structure, reliability, utility in specific populations, and confounding by general psychological distress [3, 66–72]. In particular, the original three-factor structure of the TAS-20 (consisting of DIF, DDF, and EOT) often fails to achieve adequate model fit, although the use of a bifactor structure and/or removal of reverse-coded items may alleviate this issue [3, 67, 72–74]. Most of the psychometric problems associated with the TAS-20 are driven by the EOT subscale, which often exhibits subpar internal consistency (including in the autistic sample reported by Samson et al. [65]). This subscale contains several items that relate poorly to the overall construct in certain samples, and certain EOT items seem to be particularly problematic when the scale is used in samples of children and adolescents [3, 66, 68, 69, 75].

Another issue raised in the literature is the relatively high correlation between TAS-20 scores and trait neuroticism/general psychological distress [3, 70, 71]. Although the creators of the TAS-20 have argued that the relationship between alexithymia and neuroticism is in line with theoretical predictions [3], interview measures of alexithymia such as the Toronto Structured Interview for Alexithymia (TSIA [76]) do not correlate highly with neuroticism, potentially indicating that the previously observed correlation between TAS-20 scores and neuroticism reflects a response bias on self-report items rather than the a true relationship between neuroticism and the alexithymia construct [77, 78]. Regardless of the true nature of this relationship, a high correlation between the TAS-20 and neuroticism remains problematic when not controlled for, as a sizable portion of the ability of the TAS-20 score to predict various clinical outcomes may be driven by neuroticism, which is itself a strong predictor of a number of different psychopathologies [79–82]. Notably, given the paucity of alexithymia measurement studies in samples of autistic individuals, no study to date has determined whether the TAS-20 continues to exhibit these same measurement issues in the autistic population.

Another major psychometric issue that has yet to be addressed in the alexithymia literature is the comparability of item responses between autistic and neurotypical respondents. Differential item functioning (referred to here as “item DIF” [I-DIF] to avoid confusion with the DIF TAS-20 subscale) is often present when comparing questionnaire scores between autistic and non-autistic individuals [83–85], indicating differences in the ways item responses relate to underlying traits (i.e., certain response options may be more easily endorsed at lower trait levels in one group). In cases where I-DIF is present, an autistic and neurotypicals with the same “true” alexithymia levels could systematically differ in their observed scores, resulting in incorrect conclusions about the rank order of alexithymia scores in a given sample. Moreover, I-DIF analyses test whether differences in observed scores between multiple groups (e.g., autistic and neurotypical adults) can be explained solely by group differences on the latent trait of interest or whether some trait-irrelevant factor is systematically biasing item scores in one direction or the other for a specific group. I-DIF is important to consider when comparing test scores between groups, as it has the potential to obscure the magnitude of existing group differences, either creating artifactual group differences when none exist or masking small but meaningful differences between two groups [86–88].

Although the large differences between autistic and neurotypical individuals on measures of alexithymia are unlikely to be entirely due to I-DIF, it remains possible that I-DIF may substantially bias between-group effect sizes in either direction. Furthermore, previous investigations of measurement invariance of the TAS-20 between general population samples and clinical samples of psychiatric patients have often only found evidence for partial invariance across groups [3], suggesting that I-DIF likely exists between autistic and non-autistic adults on at least some of the TAS-20 items. I-DIF may also exist between specific subgroups of the autistic population (e.g., based on age, sex, education level, or presence of comorbidities), and explicit testing of this psychometric property is necessary to determine whether a given measure can be considered equivalent across multiple sociodemographic categories. Notably, while the I-DIF null hypothesis of complete equivalence of all parameters between groups is always false at the population level [89], the effects of I-DIF may be small enough to be practically ignorable, allowing for reasonably accurate between-group comparisons [90, 91]. Thus, an important step of I-DIF analysis is the calculation of effect sizes, which help to determine whether the observed I-DIF is large enough to bias item or scales scores to a practically meaningful extent (cf. [88]).

Given the importance of the alexithymia construct in the autism literature and the many unanswered questions regarding the adequacy of the TAS-20 in multiple populations, there is a substantial need to determine whether the TAS-20 is an adequate measure of alexithymia in the autistic population. Thus, in the current study, we comprehensively evaluated the psychometric properties of the TAS-20 in a large sample of autistic adults, assessing the measure’s latent structure, reliability, and differential item functioning by diagnosis and across multiple subgroups of the autistic population. Additionally, as a secondary aim, we sought to remove poorly fitting items and items exhibiting I-DIF by diagnosis, thereby selecting a subset of the TAS-20 items that could be used to calculate a “general alexithymia” score with strong psychometric properties and the ability to accurately reflect true latent trait differences between autistic and non-autistic adults. We further established the nomological validity of the novel alexithymia score by confirming hypothesized relationships with core autism features, co-occurring psychopathology, trait neuroticism, demographic features, and quality of life. Lastly, in order to more fully interrogate the relationships between trait neuroticism and alexithymia in the autistic population, we conducted additional analyses to determine whether the novel alexithymia score was able to predict additional variance in autism features, psychopathology, and quality of life once controlling for levels of neuroticism.

## Methods

The current investigation was a secondary data analysis of TAS-20 responses collected as a part of multiple online survey studies (see “Participants” section for more details on each study). Participants reporting professional diagnoses of autism spectrum disorder were recruited from the Simons Foundation Powering Autism Research for Knowledge (SPARK) cohort, a US-based online community that allows autistic individuals and their families to participate in autism research studies [92]. In order to compare TAS scores and item responses between autistic and non-autistic individuals, we combined the SPARK sample with open data from the Human Penguin Project [93, 94], a large multinational survey study investigating the relationships between core body temperature, social network structure, and a number of other variables (including alexithymia measured using items from the TAS-20) in adults from the general population. The addition of a control group provides a substantial amount of additional information, allowing us to assess I-DIF across diagnostic groups, assess the psychometric properties of any novel alexithymia scores in the general population, and generate normative values for these scores based on the distribution of TAS-20 item responses in this sample. Although autism status was not assessed in the control sample, the general population prevalence of approximately 2% autistic adults [95] does not cause enough “diagnostic noise” in an otherwise non-autistic sample to meaningfully bias item parameter estimates or alter tests of differential item functioning [83].

### Participants

#### SPARK (autism) sample

Using the SPARK Research Match service, we invited autistic adults between the ages of 18 and 45 years to take place in our study via the SPARK research portal. All individuals self-reported a prior professional diagnosis of autism spectrum disorder or equivalent condition (e.g., Asperger syndrome, PDD-NOS). Notably, although these diagnoses are not independently validated by SPARK, the majority of participants are recruited from university autism clinics and thus have a very high likelihood of valid autism diagnosis [92]. Furthermore, validation of diagnoses in the Interactive Autism Network, a similar participant pool now incorporated into SPARK, found that 98% of registry participants were able to produce valid clinical documentation of self-reported diagnoses when requested [96]. Autistic participants in our study completed a series of surveys via the SPARK platform that included the TAS-20, additionally providing demographics, current and lifetime psychiatric diagnoses, and scores on self-report questionnaires measuring autism severity, quality of life, co-occurring psychiatric symptoms, and a number of other clinical variables (see “Measures” section for descriptions of the questionnaires analyzed in the current study). These data were collected during winter and spring of 2019 as part of a larger study on repetitive thinking in autistic adults (project number RM0030Gotham), and the SPARK participants in the current study overlap with those described by Williams et al. in several prior studies [83, 88, 97, 98]. Participants received a total of $50 in Amazon gift cards for completion of the study. A total of 1012 individuals enrolled in the study, 743 of whom were included in the current analyses. Participants were excluded if they (a) did not self-report a professional diagnosis of autism on the demographics form, (b) did not complete the TAS-20, (c) indicated careless responding as determined by incorrect answers to two instructed-response items (e.g., *Please respond *“*Strongly Agree*”* to this question*.), or (d) answered “Yes” or “Suspected” to a question regarding being diagnosed with Alzheimer’s disease (which given the age of participants in our study almost certainly indicated random or careless responding). All participants gave informed consent, and all study procedures were approved by the institutional review board at Vanderbilt University Medical Center.

#### Human Penguin Project (general population) sample

Data from a general population control sample were derived from an open dataset generated from the Human Penguin Project (HPP) [93, 94], a multinational survey study designed to test the theory of social thermoregulation [99]. Because the full details of this sample have been reported elsewhere [93, 94], we provide only a brief overview, focusing primarily on the participants whose data were utilized in the current study. The HPP sample was collected in two separate studies in 2015–2016: one online pilot study (*N* = 232) that recruited participants from Amazon’s Mechanical Turk and the similar crowdsourcing platform Prolific Academic [100, 101] and a larger cross-national study (12 countries, total *N* = 1523) that recruited subjects from 15 separate university-based research groups. In order to eliminate problems due to the non-equivalence of TAS-20 items in different languages, we used only those data where the TAS-20 items were administered in English (i.e., all crowdsourced pilot data, as well as cross-national data from the University of Oxford, Virginia Commonwealth University, University of Southampton, Singapore Management University, and University of California, Santa Barbara). Additionally, in order to match the HPP and SPARK samples on mean age, we excluded all HPP participants over the age of 60. Notably, individuals aged 45–60 were included due to the relative excess of individuals aged 20–30 in the HPP sample, which caused the subsample of 18–45-year-old HPP participants to be several years younger on average than the SPARK sample. The final HPP sample thus consisted of a total of 721 English-speaking adults aged 18–60 (MTurk *n* = 122; Prolific *n* = 84; Oxford *n* = 129; Virginia *n* = 148; Southampton *n* = 6; Singapore *n* = 132; Santa Barbara *n* = 100). As a part of this study, all participants completed 16 of the TAS-20 items, excluding four items (16, 17, 18, and 20) on the basis of poor factor loadings in the psychometric study of Kooiman et al. [66]. In addition to item-level data from these 16 TAS-20 items, we extracted the following variables: age (calculated from birth year), sex, and site of recruitment. The HPP was approved under an “umbrella” ethics proposal at Vrije Universiteit, Amsterdam, and separately at each contributing site. All study procedures complied with the ethics code outlined in the Declaration of Helsinki.

### Measures

#### Twenty-item Toronto Alexithymia Scale (TAS-20)

The TAS-20 [3, 34] is the most frequently and widely used self-report measure of alexithymia, as well as the most commonly administered alexithymia measure in the autism literature [4]. This self-report questionnaire has been used in medical, psychiatric, and general population samples as a composite measure of alexithymia for over 25 years [3], and it has been translated into over 30 languages/dialects. The TAS-20 contains twenty items rated on five-point Likert scale items from *Strongly Disagree* to *Strongly Agree*. The TAS-20 is organized into three subscales, Difficulty Identifying Feelings (DIF; 7 items), Difficulty Describing Feelings (DDF; 5 items), and Externally oriented Thinking (EOT; 8 items), corresponding to three of the four components of the alexithymia construct defined by Nemiah, Freyberger, and Sifneos [2]. Notably, the fourth component, Difficulty Fantasizing (DFAN), was also included in the original 26-item version of the TAS [35], but this subscale showed poor coherency with the other three and was ultimately dropped from the measure [3]. The sum of all 20 items on the TAS-20 is often used as an overall measure of “general alexithymia,” in line with results from several bifactor models of this questionnaire that support this interpretation [73, 74]. TAS-20 total scores of 61 or higher are typically used to create binary alexithymia classifications in both general population and clinical samples.

As noted earlier, neurotypical participants in the HPP sample filled out only 16 of the TAS-20 items, leaving out four items that demonstrated low communalities in a prior factor-analytic study [66]. However, as we wished to compare total scores from the TAS-20 between HPP and SPARK samples, we conducted single imputation for missing items in both groups using a random forest algorithm implemented in the R *missForest* package [102–104]. Such item-level imputation allowed for us to approximate the TAS-20 score distribution of the HPP participants, including the proportion of individuals exceeding the “high alexithymia” cutoff of 61. Notably, although the “high alexithymia” cutoff is theoretically questionable given the taxometric evidence for alexithymia as a purely dimensional construct [3], we chose to calculate this measure to facilitate comparisons with prior literature that primarily reported the proportion of autistic adults exceeding this cutoff [4]. To further validate the group comparisons derived from these imputed data, we additionally calculated prorated TAS-20 total scores by taking the mean of all 16 TAS-20 items administered to all participants, which was subsequently multiplied by 20 for comparability with the TAS-20 total score. These scores were then compared between groups, and the proportion of individuals in each group with prorated scores ≥ 61 was also compared to the proportions derived from (imputed) TAS-20 scores.

#### Clinical measures for validity testing

In addition to the TAS-20, individuals in the SPARK sample completed a number of other self-report questionnaires, including measures of autism symptomatology, co-occurring psychopathology, trait neuroticism, and autism-related quality of life. Measures of autistic traits included the Social Responsiveness Scale—Second Edition (SRS-2) total T-score [105] and a self-report version of the Repetitive Behavior Scale—Revised (RBS-R) [106, 107], from which we derived measures of “lower-order” and “higher-order” repetitive behaviors (i.e., the Sensory Motor [SM] and Ritualistic/Sameness [RS] subscales reported by McDermott et al. [106]). Depression was measured using autism-specific scores on the Beck Depression Inventory-II (BDI-II) [83, 108], and we additionally used BDI-II item 9 (*Suicidal Thoughts or Wishes*) to quantify current suicidality. We additionally assessed generalized and social anxiety using the Generalized Anxiety Disorder-7 (GAD-7) [109] and Brief Fear of Negative Evaluation Scale—Short Form (BFNE-S) [110, 111], respectively. Somatization was quantified using a modified version of the Patient Health Questionnaire-15 (PHQ-15) [97, 112], which extended the symptom recall period to 3 months and excluded the two symptoms of dyspareunia and menstrual problems. We measured trait neuroticism using ten items from the international personality item pool [113], originally from the Multidimensional Personality Questionnaire’s “Stress Reaction” subscale [114] and referred to here as the IPIP-N10. Lastly, general quality of life was measured using four items from the World Health Organization Quality of Life—BREF questionnaire (WHOQOL-4) [88]. More in-depth descriptions of all measures analyzed in the current study, including reliability estimates in the SPARK sample, can be found in Additional file 1: Methods.

### Statistical analyses

#### Confirmatory factor analysis and model-based bifactor coefficients

All statistical analyses were performed in the R statistical computing environment [115].

In order to test the appropriateness of the proposed TAS-20 factor structure in autistic adults, we performed a confirmatory factor analysis (CFA) on TAS-20 item responses in our SPARK sample. The measurement model in our CFA included a bifactor structure with one “general alexithymia” factor onto which all items loaded, as well as four “specific” factors representing the three subscales of the TAS-20 and the common method factor for the reverse-coded items [72]. In addition, given the previously identified problems with the EOT subscale and the reverse-coded items [3], we additionally examined a bifactor model fit only to the forward-coded DIF and DDF items, removing both the EOT and reverse-coded items. Although not the focus of the current investigation, we also fit the original and reduced TAS-20 factor models in the HPP sample in order to determine whether any identified model misfit was present only in autistic adults or more generally across both samples. Moreover, the inclusion of the HPP sample allowed for us to investigate the invariance of our reduced model across diagnostic groups, allowing us to flag items that were differentially related to the alexithymia construct in autistic and non-autistic adults. We fit the model using a diagonally weighted least squares estimator [116] with a mean- and variance-corrected test statistic (i.e., “WLSMV” estimation), as implemented in the R package *lavaan* [117]. Very few of the item responses in our dataset contained missing values (0.16% missing item responses in the SPARK sample, no missing TAS-20 data in HPP sample for the 16 administered items), and missing values were singly imputed using *missForest* [102–104].

Model fit was evaluated using the Chi-square test of exact fit, comparative fit index (CFI; 118), Tucker–Lewis index (TLI; 119), root mean square error of approximation (RMSEA; 120), standardized root mean square residual (SRMR; 121), and weighted root mean square residual (WRMR; 122, 123). The categorical maximum likelihood (cML) estimator proposed by Savalei [124] was used to calculate the CFI, TLI, and RMSEA, as these indices better approximate the population values of the maximum likelihood-based fit indices used in linear CFA than analogous measures calculated from the WLSMV test statistic [125]. Moreover, the SRMR was calculated using the unbiased estimator (i.e., SRMR_u_) proposed by Maydeu-Olivares (126, see also 127) and implemented in *lavaan* for categorical estimators. CFI_cML_/TLI_cML_ values greater than 0.95, RMSEA_cML_ values less than 0.06, SRMR_u_ values less than 0.08, and WRMR values less than 1.0 were defined as indicating adequate global model fit, based on standard rules of thumb employed in the structural equation modeling literature [121–123]. In addition to the aforementioned global fit indices, we checked for localized areas of model misfit based on examination of the residual correlations [128], with residuals greater than 0.1 indicating areas of potentially significant misfit and/or violations of local independence [129].

Confirmatory bifactor models were further interrogated with the calculation of several model-based coefficients [130–132] including (a) coefficient omega total (*ω*_T_), a measure of the reliability of the multidimensional TAS-20 total score, (b) coefficient omega hierarchical (*ω*_H_), a measure of general factor saturation (i.e., the proportion of total score variance attributable to the general factor), (c) coefficient omega subscale (*ω*_S_), a measure of the reliability for each individual subscale, (d) coefficient omega hierarchical subscale (*ω*_HS_), a measure of the proportion of subscale variance attributable to the specific factor, (e) the explained common variance (ECV; the ratio of general factor variance to group factor variance) for the total score and each item separately, and (f) the percentage of uncontaminated correlations (PUC), a supplementary index used in tandem with total ECV to determine whether a scale can be considered “essentially unidimensional” [131, 133]. Omega coefficients calculated in the current study were based on the categorical data estimator proposed by Green and Yang [134]. ECV coefficients were also calculated for individual subscales (S*-*ECV) as an additional measure of subscale general factor saturation.

#### Item response theory and differential item functioning analyses

After selecting an appropriate factor model, we evaluated the ECV and PUC coefficients to determine whether the model could be reasonably well approximated by a unidimensional item response theory (IRT) model. We then fit the data from the TAS-20 items included in the best-fitting factor model to a graded response model [135] in our SPARK sample using maximum marginal likelihood estimation [136], as implemented in the *mirt* R package [137]. Model fit was assessed using the limited-information *C*_2_ statistic [138, 139], as well as *C*_*2*_-based approximate fit indices and SRMR. Based on previously published guidelines [140], we defined values of CFI_C2_ > 0.975, RMSEA_C2_ < 0.089, and SRMR < 0.05 as indicative of good model fit. Residual correlations were examined to determine areas of local dependence, with values greater than ± 0.1 indicative of potential misfit. Items with multiple large residual correlations were flagged for removal, and the IRT model was then re-fit and iteratively tested until all areas of local misfit were removed.

After refining the unidimensional “general alexithymia” model in the SPARK sample, we further investigated the same model in the HPP sample. Once a structural model was found to fit in both samples, we fit a multi-group graded response model to the full dataset, using this model to examine I-DIF between groups. I-DIF was tested using a version of the iterative Wald procedure proposed by Cao et al. [141] and implemented in R by the first author [142], using the Oakes identity approximation method to calculate standard errors [143–145]. The Benjamini–Hochberg [146] false discovery rate (FDR) correction was applied to all omnibus Wald tests, and only those with *p*_FDR_ < 0.05 were flagged as demonstrating significant I-DIF. Significant omnibus Wald tests were followed up with tests of individual item parameters to determine which parameters significantly differed between groups [147]. Notably, this I-DIF procedure is quite powerful in large sample sizes, potentially revealing trivial group differences, and thus I-DIF effect size indices were used to determine whether the differential functioning of a given item was small enough to be ignorable in practice. In particular, we used the weighted area between curves (wABC) as a measure of I-DIF magnitude, with values greater than 0.30 indicative of practically significant I-DIF [91]. We additionally reported the expected score standardized difference (ESSD), a standardized effect size interpretable on the metric of Cohen’s *d* [90]. Items exhibiting practically significant I-DIF between autistic and non-autistic adults were further flagged for removal, and this process was repeated iteratively until none of the resulting set of items displayed practically significant I-DIF by diagnostic group. The total effect of all I-DIF (i.e., differential test functioning [DTF]) was then estimated using the unsigned expected test score difference in the sample (UETSDS), the expected absolute difference in manifest test scores between individuals of different groups possessing the same underlying trait level [91].

After removing items based on between-group I-DIF, we then examined I-DIF of the resulting short form across subsets of the autistic population. Using the same iterative Wald procedure and effect size criteria as the between-group analyses, we tested whether TAS items functioned differently across groups based on sex, gender, age (> 30 vs. ≤ 30 years), race (non-Hispanic White vs. Other), level of education (any higher education vs. no higher education), age of autism diagnosis (≥ 18 years old vs. < 18 years), self-reported co-occurring conditions (current depressive disorder, current anxiety disorder, and lifetime attention deficit hyperactivity disorder [ADHD]). Although many fewer stratification variables were collected in the HPP sample, I-DIF was also examined within that sample according to age (> 30 vs. ≤ 30 years), sex, and phase of the project (i.e., pilot study vs. multi-site study). These I-DIF results were used to further confirm that the resulting general alexithymia factor score exhibited I-DIF across all groups that was small enough to be practically ignorable. All items retained at this stage were incorporated into the final general alexithymia factor score.

Once the item set for the general alexithymia factor score was finalized, we then fit an additional multi-group graded response model on only those final items, constraining item parameters to be equal between groups and setting the scale of the latent variable by constraining the general population sample to have a mean of 0 and standard deviation of 1. Using this model, we then estimated maximum a posteriori (MAP) latent trait scores for each individual, which were interpretable as Z-scores relative to the general population (i.e., a score of 1 is one full standard deviation above the mean of our non-autistic normative sample). Individual reliability coefficients were also examined, with values greater than 0.7 being deemed sufficiently reliable for interpretation at the individual level.

#### Validity testing

To further test the validity of the novel general alexithymia latent trait scores in autistic adults, we investigated the relationships between these scores and a number of clinical variables that have previously demonstrated relationships with alexithymia in either autistic adults or the general population. Based on previous literature [60], we hypothesized that alexithymia would show moderate-to-strong positive correlations with neuroticism (IPIP-N10), autistic traits (SRS-2), repetitive behavior (RBS-R), depression (BDI-II), generalized anxiety (GAD-7), social anxiety (BFNE-S), suicidality (BDI item 9), and somatic symptom burden (PHQ-15), as well as moderate negative correlations with autism-specific QoL (WHOQOL-4). Given the documented relationships between neuroticism and alexithymia, we further examined the magnitude of these correlations after controlling for levels of neuroticism. We additionally examined relationships between alexithymia scores and demographic variables, including age, sex, race/ethnicity, age of autism diagnosis, and level of education. Notably, alexithymia is correlated with older age, male sex, and lower education level in the general population [148–150], and we expected that these relationships would replicate in the current SPARK sample (with the exception of the correlation with age, given the restricted age range in our current sample). We did not, however, expect to find significant associations between alexithymia and race/ethnicity or age of autism diagnosis.

Relationships between alexithymia and external variables were examined using robust Bayesian variants of the Pearson correlation coefficient (for continuous variables, e.g., SRS-2 scores), polyserial correlation coefficient (for ordinal variables, such as the BDI-II suicidality item and education level), partial correlation coefficient (when testing relationships after controlling for neuroticism), and unequal-variances *t* test [151–153], as implemented using custom R code [154] and the *brms* package [155]. Additional technical details regarding model estimation procedures and prior distributions can be found in Additional file 1: Methods. Standardized effect sizes produced by these methods (i.e., *r*, *r*_p_, and *d*) were summarized using the posterior median and 95% highest-density credible interval (CrI). Zero-order correlations with psychopathological variables of interest were also repeated using the TAS-20 total score to investigate the degree to which the revised alexithymia score maintained the nomological validity of the longer measure.

In addition to estimating the magnitude of each effect size, we tested these effects for “practical significance” [156] within a Bayesian hypothesis testing framework. To do this, we defined *interval* null hypotheses within which all effect sizes were deemed too small to be practically meaningful. This interval, termed the region of practical equivalence (ROPE) [157], was defined in the current study as the interval *d* = [− 0.2, 0.2] for *t* tests, *r* = [− 0.2, 0.2] for bivariate correlations, and *r*_p_ = [− 0.1, 0.1] for partial correlations. Evidence both *for or against* this interval null hypothesis can be quantified by calculating the ROPE Bayes factor (*BF*_ROPE_), which is defined as the odds of the prior effect size distribution falling within the ROPE divided by the odds of the posterior effect size distribution falling within the ROPE [158, 159]. In accordance with standard interpretation of Bayes factor values [160, 161], we defined *BF*_ROPE_ values greater than 3 as providing substantial evidence for ${\mathcal{H}}_{1}$ (i.e., the true population effect lies outside the ROPE) and *BF*_ROPE_ values less than 0.333 as providing substantial evidence for ${\mathcal{H}}_{0}$ (i.e., the true population effect lies within the ROPE and thus is not practically meaningful). Values of *BF*_ROPE_ between 0.333 and 3 are typically considered inconclusive, providing only “anecdotal” evidence for either ${\mathcal{H}}_{0}$ or ${\mathcal{H}}_{1}$ [160].

#### Readability analysis

As a supplemental analysis, we evaluated the readability of the TAS-20 and the newly derived short form using the FORCAST formula [162]. This formula is well suited for questionnaire material, as it ignores the number of sentences, average sentence length, or hard punctuation (standard metrics for text in prose form), instead of focusing exclusively on the number of monosyllabic words [163]. FORCAST grade level equivalent was calculated for both the TAS-20 (excluding the questionnaire directions) and the set of items contributing to the general alexithymia factor derived in the current study. Additionally, in order to compare our results with prior work on the readability of the TAS-20, we calculated the Flesch–Kincaid grade level (FKGL) and Flesch Reading Ease (FRE) scores [164, 165] for both the TAS-20 and general factor items. All readability analyses were conducted using Readability Studio version 2019.3 (Oleander Software, Ltd, Vandalia, OH, USA). Although we did not attempt to select items based on readability, this analysis was constructed to ensure that the newly selected subset of items did not have a substantially higher reading level, which would indicate that younger or less educated respondents may produce scores of questionable validity.

## Results

### Participants and demographics

In total, our sample included TAS data from 1464 unique individuals across the two data sources (Table 1). Autistic adults in the SPARK sample (*n* = 743, age = 30.91 ± 7.02 years, 63.5% female sex) were predominantly non-Hispanic White (79.4%) and college-educated (46.4% with a 2- or 4-year college degree, and an additional 26.5% with some college but no degree), similar to the previous sample drawn from this same SPARK project [83]. The median age of autism diagnosis was 19.17 years (IQR = [10.33, 28.79]), indicating the majority of individuals in the sample were diagnosed in adulthood. Most autistic participants reported a current depressive or anxiety disorder (defined as symptoms in the past 3 months or an individual currently being treated for one of these disorders), with depression present in 59.2% and anxiety present in 71.7%. TAS-20 scores in the SPARK sample were present across the full range of trait levels (*M* = 60.55, SD = 13.11), and just over half of the sample (54.5%) was classified as “high alexithymia” based on TAS-20 total scores greater than or equal to 61. Less demographic information was available for the general population adults in the HPP sample (*n* = 721, age = 30.92 ± 13.01 years, 64.9% female), but the available demographics indicated that these individuals were well matched to the SPARK sample on age and sex. Partially imputed TAS-20 scores in the HPP sample were slightly higher than other general population samples (*M* = 50.21, SD = 11.21), and based on these scores, 17.1% of HPP participants were classified as having “high alexithymia.” Prorated TAS-20 total scores in the HPP sample (*M* = 51.38, SD = 10.92) were similar in magnitude to the imputed TAS-20 scores, with a slightly larger proportion of the HPP sample (19.1%) classified as “high alexithymia” using this method. As anticipated, large differences in both TAS-20 total scores (*d* = 0.880, 95% CrI [0.767, 0.995]) and prorated TAS-20 total scores (*d* = 0.811, 95% CrI [0.697, 0.922]) were present between groups.

**Table 1:** Demographics for autistic and general population samples

	SPARK (*n* = 743)	HPP (*n* = 721)
Age (years)	30.91 (7.02)	30.92 (13.01)
Sex		
Male	271 (36.5%)	253 (35.1%)
Female	472 (63.5%)	468 (64.9%)
Gender identity		
Cisgender man	245 (33.0%)	–
Cisgender woman	400 (53.8%)	–
Transgender man	15 (2.0%)	–
Transgender woman	6 (0.8%)	–
Non-binary	76 (10.2%)	–
Non-Hispanic White	590 (79.4%)	–
Education		
No high school diploma	25 (3.4%)	–
High school diploma/GED	140 (18.8%)	–
Vocational certificate	36 (4.8%)	–
Some college	197 (26.5%)	–
Associate degree	74 (10.0%)	–
Bachelor’s degree	171 (23.0%)	–
Graduate/professional degree	100 (13.5%)	–
Age of autism diagnosis (years)	19.67 (11.17)	–
Current depression	440 (59.2%)	–
Current anxiety	533 (71.7%)	–
Current suicidality	292 (39.3%)	–
Lifetime ADHD	342 (46.0%)	–
TAS-20 total score	60.55 (13.11)	50.21 (11.21)^a^
TAS-20 total score (prorated)^b^	61.26 (14.17)	51.38 (10.92)
GAFS-8 latent trait score	1.01 (1.17)	0.01 (0.93)
“High alexithymia” (TAS-20 ≥ 61)	405 (54.5%)	123 (17.1%)^a^

Continuous variables are presented as *M* (*SD*), and categorical variables are presented as *N* (%). All data in both samples were gathered by self-report

*SPARK* Simons Powering Autism Research Knowledge, *HPP* Human Penguin Project, *ADHD* attention deficit hyperactivity disorder, *TAS* Toronto Alexithymia Scale, *GAFS-8* 8-item General Alexithymia Factor Score

^a^Participants in the HPP sample completed 16 items of the TAS-20, which excluded items 16, 17, 18, and 20. For comparison with the TAS-20 scores in the SPARK sample, these four items were imputed for all HPP participants using random forest imputation

^b^Calculated as mean of all 16 non-missing TAS-20 items multiplied by 20, for comparison with TAS-20 scores

### Confirmatory factor analysis

Within the SPARK sample, the confirmatory factor model for the full TAS-20 exhibited subpar model fit, with only the SRMR_u_ meeting a priori fit index cutoff values (Table 2). Additionally, examination of residual correlations revealed five values greater than 0.1, indicating a non-ignorable degree of local model misfit. Model-based bifactor coefficients indicated strong reliability and general factor saturation of the TAS-20 composite (*ω*_T_ = 0.912, *ω*_H_ = 0.773), though the ECV/PUC indicated that the scale could not be considered “essentially unidimensional” (ECV = 0.635, PUC = 66.8%). Both the DIF and DDF subscales exhibited good composite score reliability (*ω*_S_ = 0.906 and 0.854, respectively), although omega hierarchical coefficients indicated that the vast majority of reliable variance in each subscale was due to the “general alexithymia” factor (DIF: *ω*_HS_ = 0.162, S-ECV = 0.753; DDF: *ω*_HS_ = 0.145, S-ECV = 0.768, respectively). Conversely, the EOT subscale exhibited very poor reliability, with only one fourth of common subscale variance attributable to the general factor (*ω*_S_ = 0.451, *ω*_HS_ = 0.300, S-ECV = 0.245). Examination of the factor loadings further confirmed the inadequacy of the EOT subscale, as seven of the eight EOT items (5, 8, 10, 15, 16, 18, 19, and 20) loaded poorly onto the “general alexithymia” factor (*λ*_G_ =  − 0.116 to 0.311; Additional file 1: Table S1). Notably, these psychometric issues were not limited to autistic adults. The fit of the TAS-20 CFA model in the HPP sample was equally poor, and bifactor coefficients indicating the psychometric inadequacy of the EOT and reverse-scored items were replicated in this sample as well (Table 2).

**Table 2:** Confirmatory factor analysis fit indices and model-based omega coefficients

Index	TAS-20 Bifactor: SPARK	TAS-20 Bifactor: HPP	11-item Bifactor: SPARK	11-item Bifactor: HPP
*Model FIT INDICEs*				
*χ*^2^ (*df*)^a^	590.6 (145)	669.9 (145)	151.6 (33)	124.0 (33)
CFI_cML_	0.924	0.900	**0.970**	**0.978**
TLI_cML_	0.900	0.869	**0.951**	**0.963**
RMSEA_cML_ [90% CI]	0.072 [0.066, 0.078]	0.086 [0.081, 0.092]	0.080 [0.069, 0.092]	0.068 [**0.056**, 0.079]
SRMR_u_ [90% CI]	**0.036 [0.033, 0.004]**	**0.051 [0.047, 0.056]**	**0.020 [0.017, 0.024]**	**0.019 [00.015, 0.023]**
WRMR	1.119	1.565	**0.768**	**0.699**
|Residuals| > 0.1	2.60%	8.90%	**0%**	**0%**
Largest residual	0.149	0.225	**0.084**	**0.055**
*Bifactor coefficients*				
*ω*_T_/*ω*_H_	0.912/0.773	0.914/0.741	0.929/0.861	0.925/0.952
*ω*_S_/*ω*_HS_ (DIF)	0.906/0.162	0.880/0.224	0.913/0.087	0.892/0.071
*ω*_S_/*ω*_HS_ (DDF)	0.854/0.145	0.803/0.120	0.800/0.163	0.839/0.223
*ω*_S_/*ω*_HS_ (EOT)	0.451/0.300	0.512/0.307	–	–
*ω*_S_/*ω*_HS_ (REV)	0.559/0.441	0.692/0.689	–	–

Fit indices that above the a priori cutoffs for acceptable model fit (CFI/TLI > 0.95, RMSEA < 0.06, SRMR < 0.08, WRMR < 1, all residuals < 0.1) are presented in bold. TAS = Toronto Alexithymia Scale; SPARK = Simons Powering Autism Research Knowledge; HPP = Human Penguin Project; CFI_cML_ = comparative fit index (categorical maximum likelihood estimation); TLI_cML_ = Tucker–Lewis Index (categorical maximum likelihood estimation); RMSEA_cML_ = root mean square error of approximation (categorical maximum likelihood estimation); SRMR_u_ = population-unbiased standardized root mean square residual; WRMR = weighted root mean square residual; *ω*_T_ = omega total (composite reliability of total score); *ω*_H_ = omega hierarchical (proportion of total score variance accounted for by general factor); *ω*_S_ = omega subscale (composite reliability of subscale score); *ω*_HS_ = omega hierarchical subscale (proportion of subscale score variance accounted for by specific factor); DIF = difficulty identifying feelings; DDF = difficulty describing feelings; EOT = externally oriented thinking; REV = reverse-coded item method factor

^a^All *p* values < 0.001

Following the removal of the EOT and reverse-coded items from the TAS-20, we fit a bifactor model with two specific factors (DIF and DDF) to the remaining 11 items in our SPARK sample. The fit of this model was substantially improved over the TAS-20, with all indices except RMSEA_cML_ exceeding a priori designated cutoffs (Table 2) and all residual correlations below 0.1. Moreover, model-based coefficients (ECV = 0.815; PUC = 50.9%) indicated that the 11-item composite was unidimensional enough to be fit by a standard graded response model with little parameter bias. Notably, the estimated reliability and general factor saturation of this 11-item composite score were higher than those of the 20-item composite (*ω*_T_ = 0.925, *ω*_H_ = 0.852), suggesting that the inclusion of EOT and reverse-coded items on the scale *reduced* the amount of total score variance attributable to the underlying “general alexithymia” construct. Fit of the 11-item unidimensional model in the HPP sample was equally strong (Table 2), with an approximately equal ECV (0.793) supporting the essential unidimensionality of this scale in both samples.

### Item response theory analyses

A unidimensional graded response model fit to the 11 remaining TAS-20 items did not display adequate fit according to a priori fit index guidelines (*C*_2_(44) = 485.7, *p* < 0.001, *CFI*_C2_ = 0.955, *RMSEA*_C2_ = 0.116, *SRMR* = 0.068). Examination of residual correlations indicated that item 7 (*I am often puzzled by sensations in my body*) was particularly problematic, exhibiting a very large residual correlation of 0.259 with item 3 as well as two other residuals greater than 0.1. Removal of this item caused the resulting 10-item graded response model to approximately meet the minimum standards for adequate fit (*C*_2_(35) = 485.7, *p* < 0.001, CFI_C2_ = 0.976, RMSEA_C2_ = 0.086, SRMR = 0.051), with all remaining residual correlations below 0.1. The overall fit of this 10-item model was somewhat worse in the HPP sample (*C*_2_(35) = 319.9, *p* < 0.001, CFI_C2_ = 0.960, RMSEA_C2_ = 0.106, SRMR = 0.065); however, it is notable that this model contained item 17, which was not administered in the HPP survey and was thus fully imputed in this sample. Removal of this item from the model resulted in a substantial improvement in fit in the HPP sample (*C*_2_(27) = 169.1, *p* < 0.001, CFI_C2_ = 0.974, RMSEA_C2_ = 0.086, SRMR = 0.058), with fit indices approximately reaching the a priori cutoffs. As the 9-item unidimensional model also exhibited good fit in the SPARK sample (C_2_(27) = 161.7, *p* < 0.001, CFI_C2_ = 0.980, RMSEA_C2_ = 0.082, SRMR = 0.049), we chose this version of the measure to test I-DIF between autistic and general population adults.

For the remaining nine TAS-20 items, I-DIF was evaluated across diagnostic groups using the iterative Wald test procedure. Significant I-DIF was found in eight of the nine items (all except item 6) at the *p* < 0.05 level (Table 3); however, effect size indices suggested that practically significant I-DIF was only present in item 3 (*I have physical sensations that even doctors don’t understand*; wABC = 0.433, ESSD = 0.670). The remaining items all exhibited I-DIF with small standardized effect sizes (all wABC < 0.165, all |ESSD| < 0.187), allowing these effects to be ignored in practice [91]. After removal of item 3, we re-tested I-DIF for the remaining eight items, producing nearly identical results (significant I-DIF for all items except 6; all wABC < 0.167, all |ESSD| < 0.186). The overall DTF of the 8-item composite was also small enough to be ignorable, with the average difference in total scores between autistic and non-autistic adults of the same trait level being less than 0.5 scale points (UETSDS = 0.460, ETSSD =  − 0.011).

**Table 3:** Differential item functioning results comparing autistic and general population adults on 9-item unidimensional model

TAS-20 Item #	χ^2^(5)	*p*_FDR_	wABC	ESSD	Parameters^a^
1	35.30	< 0.001	0.089	− 0.018	*a*_*1*_, *d*_*1*_, ***d***_***2***_
2	23.18	< 0.001	0.164	0.157	***d***_***2***_, ***d***_***3***_
3	65.10	< 0.001	0.433^b^	0.670^b^	***d***_***2***_**, *d***_***3***_**, *d***_***4***_
9	26.03	< 0.001	0.064	− 0.021	*d*_*1*_
11	30.47	< 0.001	0.165	0.001	*a*_*1*_, ***d***_***2***_**, *d***_***3***_
12	30.19	< 0.001	0.149	− 0.187	*d*_*1*_
13	57.66	< 0.001	0.064	− 0.022	*a*_*1*_, *d*_*1*_, ***d***_***2***_**, *d***_***3***_**, *d***_***4***_
14	61.90	< 0.001	0.031	− 0.022	*a*_*1*_, *d*_*1*_, ***d***_***2***_**, *d***_***3***_**, *d***_***4***_

Results indicate omnibus Wald tests of differential item functioning using the iterative anchor-selection method of Cao et al. (2017). *P* values (*p*_FDR_) are corrected for a 5% false discovery rate using the Benjamini–Hochberg procedure. Parameters that were significantly different between groups when tested alone with follow-up Wald tests (*p*_FDR_ < 0.05) are indicated in the Parameters column. TAS-20 = 20-item Toronto Alexithymia Scale; wABC = weighted area between curves; ESSD = expected score standardized difference (in Cohen’s *d* metric); *a*_*1*_ = slope parameter; *d*_*1*_*–d*_*4*_ = item intercept parameters (i.e., item “difficulty” parameters)

^a^Parameters in bold are larger (i.e., more discriminating for *a* parameters and “easier” for *d* parameters) in the autistic group. Larger values of *a* indicate that the item is more strongly related to the latent trait in autistic adults, whereas larger values of *d* indicate that a given item response is endorsed at lower latent trait levels in autistic adults relative to the general population

^b^Practically significant DIF (i.e., wABC > 0.3)

After establishing practical equivalence in item parameters between the two diagnostic groups, we then tested I-DIF for the 8-item composite for a number of subgroups within the HPP and SPARK samples. Within the general population HPP sample, all eight items displayed no significant I-DIF across by sex, age (≥ 30 vs. < 30), or phase of the HPP study (all *p*s > 0.131). Similarly, in the SPARK sample, there was no significant I-DIF by sex, gender, race, education level, current anxiety disorder, history of ADHD, or current suicidality (all *p*s > 0.105). However, significant I-DIF was found across several demographics, including age (item 6; wABC = 0.0543, ESSD =  − 0.045), age of autism diagnosis (items 2, 6, and 14; all wABC < 0.267, all |ESSD| < 0.135), and current depressive disorder (item 13; wABC = 0.274, ESSD = 0.361), although wABC values for these items indicated that the degree of I-DIF was ignorable in practice.

**Fig. 1:**
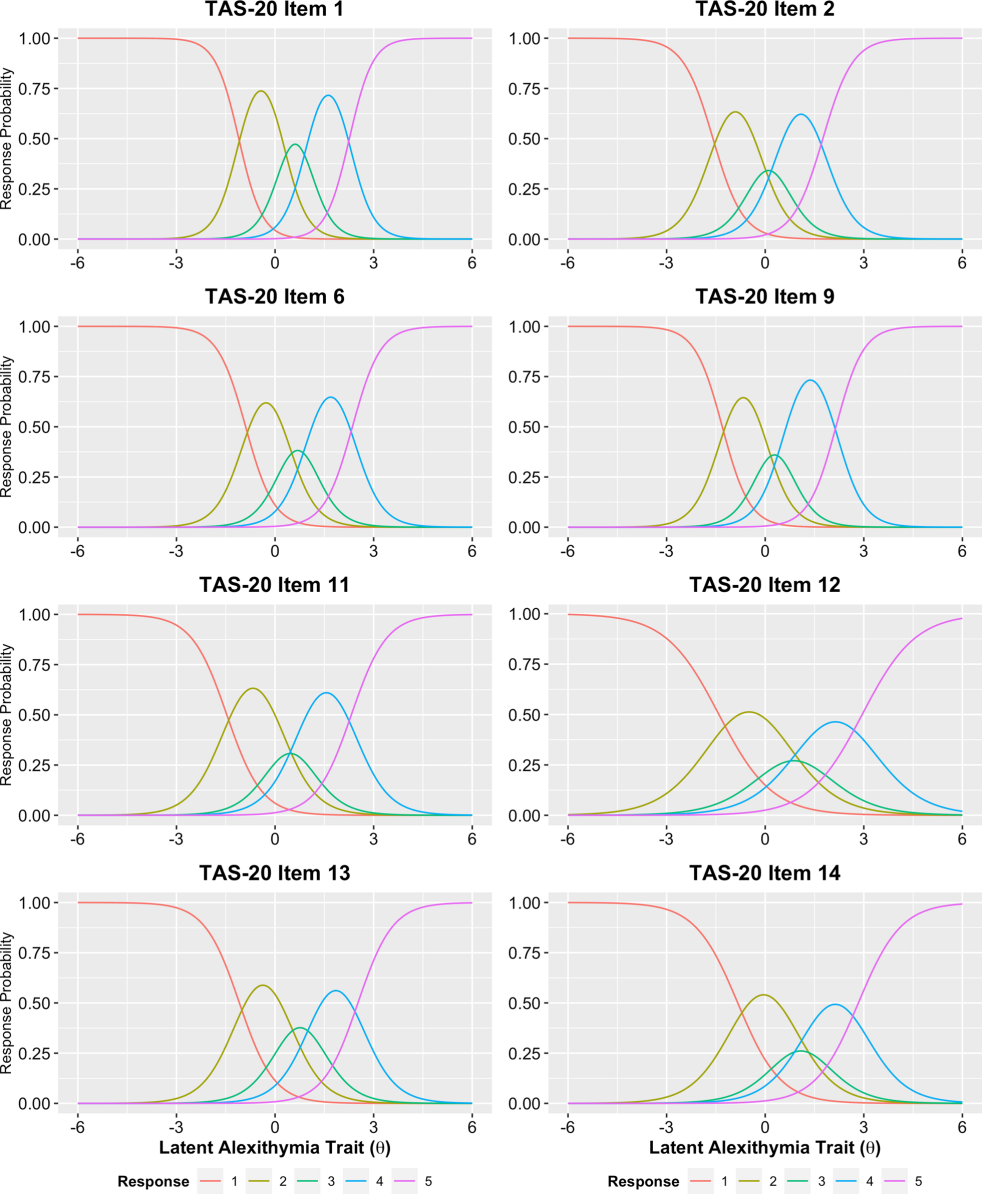
Item category characteristic curves (i.e., “trace lines”) for the eight GAFS-8 items. Three of the items (TAS-20 items 11, 12, and 14) had neutral (“3”) responses that were not the most probable response at any point along the latent trait continuum, indicating that these response options were underutilized in our combined sample

**Fig. 2:**
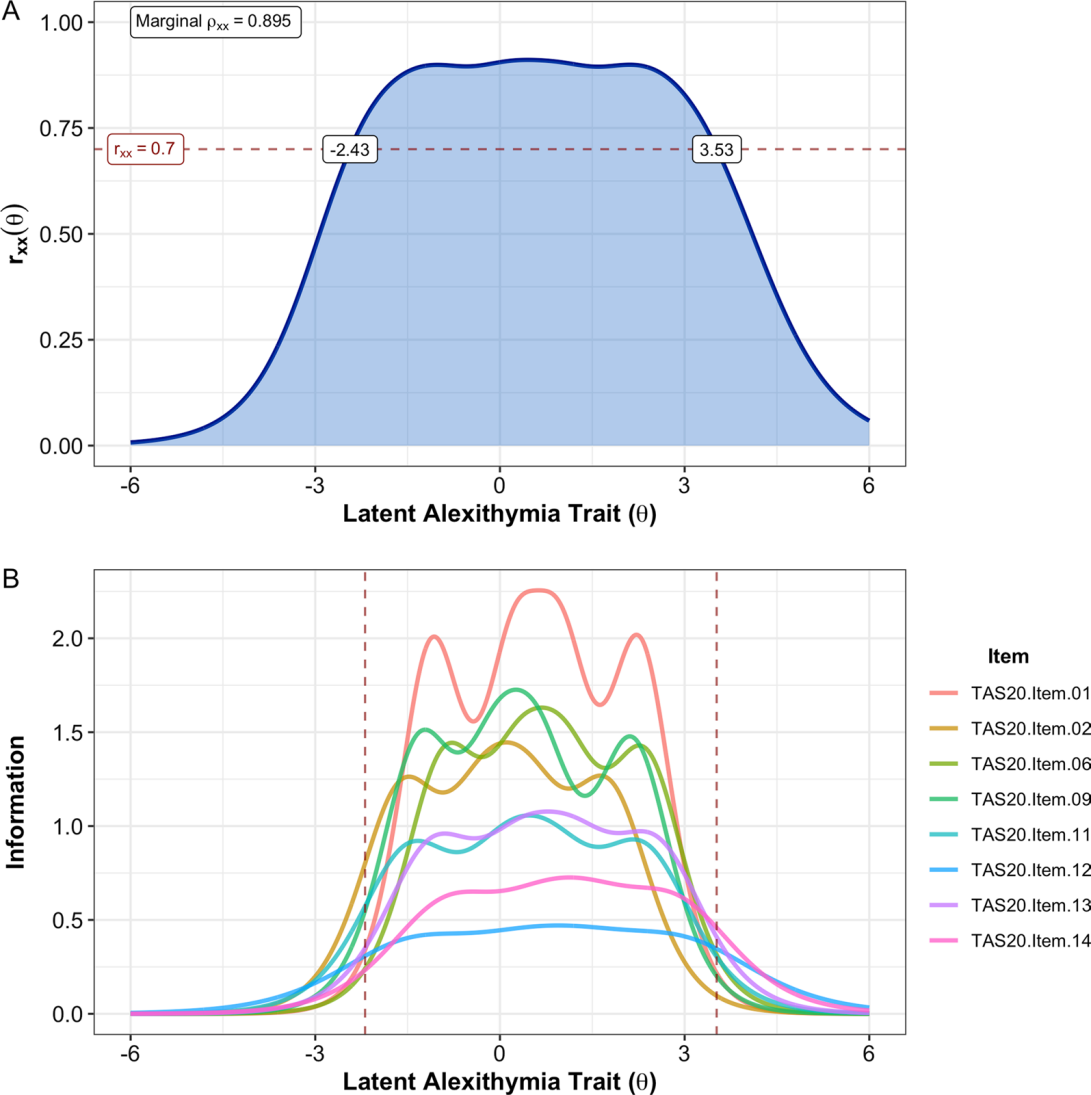
**A** Estimated reliability of GAFS-8 latent trait scores across the full latent alexithymia continuum. The horizontal dashed line indicates *r*_xx_ = 0.7, the a priori threshold for acceptable score reliability. Individual reliabilities for trait scores between − 2.43 and 3.53 are all greater than or equal to this cutoff, including all trait levels estimable by the GAFS-8 (i.e., *θ* between − 2.19 and 3.52). **B** Item-level information functions for GAFS-8 items. Vertical dashed lines indicate the trait levels captured by the minimum GAFS-8 score (all “0” responses, *θ* =  − 2.19) and the maximum GAFS-8 score (all “5” responses, *θ* = 3.52). The sum of all item information functions equals the test information function

As none of the eight retained items exhibited practically significant I-DIF across any of the tested contrasts, we retained all eight items for the final alexithymia factor score, which we termed the “8-item general alexithymia factor score” (abbreviated as GAFS-8). A graded response model fit to the GAFS-8 items in the full sample exhibited adequate fit (*C*_2_(20) = 240.4, *p* < 0.001, CFI_C2_ = 0.983, RMSEA_C2_ = 0.087, SRMR = 0.045) and no residual correlations greater than 0.1. A multi-group model with freely estimated mean/variance for the autistic group was used to calculate the final item parameters (Table 4), as well as individual latent trait scores. Item characteristic curves indicated that all GAFS-8 items behaved appropriately, although the middle response option was insufficiently utilized for three of the eight items (Fig. 1). The MAP-estimated latent trait scores for the GAFS-8 showed strong marginal reliability (*ρ*_xx_ = 0.895, 95% bootstrapped CI: [0.895, 0.916]), and individual reliabilities were greater than the minimally acceptable 0.7 for the full range of possible GAFS-8 scores (i.e., latent trait values between − 2.19 and 3.52; Fig. 2A). Item information plots for the eight GAFS-8 items (Fig. 2B) indicated that all items contributed meaningful information to the overall test along the full trait distribution of interest. GAFS-8 latent trait scores were also highly correlated with total scores on the TAS-20 (*r* = 0.910, 95% CrI [0.897, 0.922]), indicating that the general alexithymia factor being assessed by this short form is strongly related to the more established version of the general alexithymia construct reflected by the TAS-20 total score [73, 74]. Diagnostic group differences in GAFS-8 latent trait scores remained large, with autistic individuals demonstrating substantially elevated levels of alexithymia on this measure (*d* = 1.014 [0.887, 1.139]).

**Table 4:** GAFS-8 graded response model parameters and equivalent factor loadings for full sample

TAS-20 Item #	*a*_*1*_	*d*_*1*_	*d*_*2*_	*d*_*3*_	*d*_*4*_	*λ*	*h*^2^
1	2.802	3.092	− 0.689	− 2.740	− 6.336	0.855	0.731
2	2.190	3.478	0.491	− 0.931	− 3.841	0.790	0.623
6	2.335	2.090	− 0.805	− 2.413	− 5.497	0.808	0.653
9	2.402	3.137	0.072	− 1.434	− 5.170	0.816	0.666
11	1.870	2.745	− 0.234	− 1.505	− 4.340	0.740	0.547
12	1.235	1.739	− 0.526	− 1.636	− 3.644	0.587	0.345
13	1.892	2.054	− 0.646	− 2.231	− 4.771	0.743	0.553
14	1.538	1.285	− 1.133	− 2.201	− 4.361	0.671	0.450

Parameters estimated using maximum marginal likelihood based on Bock–Aitkin EM algorithm. This model contained two groups: general population (*θ* fixed to *M* = 0, *SD* = 1 in this group) and autistic group (mean and SD of *θ* free to vary), with all item parameters constrained to equality between groups. TAS-20 = 20-item Toronto Alexithymia Scale; *a*_*1*_ = slope parameter; *d*_*1*_*–d*_*4*_ = item intercept parameters (more positive values indicate “easier” items); *λ* = factor loading on single factor; *h*^2^ = communality (squared factor loading)

### Validity analyses

Overall, the GAFS-8 demonstrated a pattern of correlations with other variables that generally resembled the relationships seen with the TAS-20 total score in other clinical and non-clinical samples (Table 5). The GAFS-8 was highly correlated with autistic traits as measured by the SRS-2 (*r* = 0.642 [0.598, 0.686]), additionally exhibiting moderate correlations with lower-order (*r* = 0.386 [0.320, 0.450]) and higher-order (*r* = 0.432 [0.372, 0.494]) repetitive behaviors as measured by the RBS-R. GAFS-8 latent trait scores were also correlated with psychopathology measures, exhibiting the hypothesized pattern of correlations with depression, anxiety, somatic symptom burden, social anxiety, and suicidality (*r*s = 0.275–0.423), as well as lower general quality of life (*r* =  − 0.357 [− 0.419, − 0.291]). When examining these correlations using the TAS-20 total score in place of the GAFS-8 score (Additional file 1: Table S2), the GAFS-8 score demonstrated numerically stronger correlations with eight of ten external variables (all except SRS-2 total scores and PHQ-15 scores; Additional file 1: Table S2), though the magnitudes of these differences were very small (all |∆*r*|s < 0.07), suggesting practically equivalent correlations with external variables. As with the TAS-20 total score, the GAFS-8 displayed a moderate-to-large correlation with trait neuroticism (*r* = 0.475 [0.416, 0.531]), raising the possibility that relationships between GAFS-8 scores and internalizing psychopathology are driven by neuroticism rather than alexithymia per se. To investigate this possibility further, we calculated partial correlations between the GAFS-8 and other variables after controlling for IPIP-N10 scores, using a Bayes factor to test the interval null hypothesis that *r*_p_ falls between − 0.1 and 0.1 (i.e., < 1% of additional variance in the outcome is explained by the GAFS-8 score after accounting for neuroticism). Bayes factors provided substantial evidence that the partial correlations between the GAFS-8 and SRS-2, RBS-R subscales, and BDI-II exceeded the ROPE. Additionally, while partial correlations with the BFNE-S, PHQ-15, and BDI suicidality item were all greater than zero, Bayes factors suggested that all three of these correlations were more likely to lie within the ROPE than outside of it (all *BF*_ROPE_ < 0.258). There was only anecdotal evidence that the partial correlation between the GAFS-8 and GAD-7 exceeded the ROPE (*BF*_ROPE_ = 2.18). However, there was a 91.3% posterior probability of that correlation exceeding the ROPE, suggesting that there was a strong likelihood of alexithymia explaining a meaningful amount of additional variance in anxiety symptoms beyond that accounted for by neuroticism. Conversely, while the partial correlation between the GAFS-8 and general quality of life remained nonzero after controlling for neuroticism (*r* =  − 0.113 [− 0.188, − 0.039]), there was insufficient evidence that this relationship met our criterion for practical significance (63.3% posterior probability that *r*_p_ <  − 0.1, *BF*_ROPE_ = 0.348).

**Table 5:** Zero-order and partial correlations between GAFS-8 latent trait scores and other clinical measures in SPARK sample

Covariate	*r* [95% CrI]	*BF*_ROPE_	$P(\mathrm{ROPE}|\mathrm{Data}$)	*r*_p_ [95% CrI]	*BF*_ROPE_	$P(\mathrm{ROPE}|\mathrm{Data}$)
SRS-2	0.642 [0.598, 0.686]	**2.07 × 10**^**20**^	< 0.001	0.514 [0.458, 0.567]	**3.74 × 10**^**18**^	< 0.001
RBS-R SM	0.385 [0.322, 0.444]	**9.83 × 10**^**6**^	< 0.001	0.294 [0.225, 0.363]	**9.05 × 10**^**4**^	< 0.001
RBS-R RS	0.432 [0.372, 0.494]	**1.25 × 10**^**7**^	< 0.001	0.297 [0.228, 0.362]	**1.68 × 10**^**5**^	< 0.001
BDI-II	0.420 [0.358, 0.480]	**1.28 × 10**^**7**^	< 0.001	0.159 [0.086, 0.232]	**3.34**	0.059
GAD-7	0.423 [0.360, 0.481]	**1.34 × 10**^**7**^	< 0.001	0.150 [0.082, 0.222]	2.18	0.087
BFNE-S	0.358 [0.292, 0.423]	**3.21 × 10**^**4**^	< 0.001	0.105 [0.030, 0.180]	*0.258*	0.446
PHQ-15	0.275 [0.208, 0.346]	**23**	0.019	0.093 [0.019, 0.165]	*0.150*	0.579
WHOQOL-4	− 0.357 [− 0.419,− 0.291]	**4.23 × 10**^**4**^	< 0.001	− 0.113 [− 0.188,− 0.039]	0.348	0.367
Suicidality	0.303 [0.222, 0.382]	**19**	0.009	0.111 [0.021, 0.198]	*0.124*	0.403
IPIP-N10	0.475 [0.416, 0.531]	**9.90 × 10**^**9**^	< 0.001	–	–	–

All partial correlations (*r*_p_) control for neuroticism (IPIP-N10 scores) when examining the correlation between GAFS-8 scores and other variables of interest. Bayes factors indicating substantial evidence against the interval null hypothesis (i.e., *r* or lies within [− 0.2, 0.2] or *r*_p_ lies within [− 0.1, 0.1]) are presented in bold, whereas Bayes factors indicating substantial evidence *for* the interval null hypothesis are presented in italics. Correlations are estimated using Bayesian methods and are presented along with 95% highest-density credible intervals (CrI). *BF*_ROPE_ = Bayes factor assessing interval null hypothesis that the effect falls within the region of practical equivalence (ROPE); $P(\mathrm{ROPE}|Data$) = proportion of the *r*/*r*_p_ posterior distribution falling within the ROPE, conditioned on the observed data (i.e., probability that the interval null hypothesis is true); SRS-2 = Social Responsiveness Scale—Second Edition; RBS-R = Repetitive Behavior Scale—Revised; SM = Sensory Motor (“lower-order” repetitive behaviors) subscale; RS = Ritualistic/Sameness (“higher-order” repetitive behaviors) subscale; BDI-II = Beck Depression Inventory-II; GAD-7 = Generalized Anxiety Disorder-7; BFNE-S = Brief Fear of Negative Evaluation—Short; PHQ-15 = (modified) Patient Health Questionnaire-15; WHOQOL-4 = 4-Item World Health Organization Quality of Life Score; Suicidality = BDI-II item 9 (*Suicidal Thoughts or Wishes*); IPIP-N10 = 10-item neuroticism scale from the international personality item pool

The relationships between GAFS-8 scores and demographic variables were also examined in order to determine whether relationships found in the general population apply to autistic adults. As hypothesized, GAFS-8 scores showed a small and practically insignificant correlation with age (*r* = 0.032 [− 0.041, 0.104], *BF*_ROPE_ = 5.77 × 10^–6^), likely due to the absence of older adults (i.e., ages 60 +) in our sample. The GAFS-8 also showed a nonzero negative correlation with education level, although the magnitude of this relationship was small enough to not be practically significant (*r*_poly_ =  − 0.089 [− 0.163, − 0.017], *BF*_ROPE_ = 0.045). Unlike in the general population, females in the SPARK sample had slightly higher GAFS-8 scores (*d* = 0.183 [0.022, 0.343]), although this difference was small and not practically significant (*BF*_ROPE_ = 0.265). Additionally, there was an absence of practically significant differences in alexithymia by race/ethnicity (*d* =  − 0.052 [− 0.247, 0.141], *BF*_ROPE_ = 0.029). Lastly, age of autism diagnosis was positively correlated with GAFS-8 scores (*r* = 0.133 [0.06, 0.204]), although this correlation was also small enough to not be practically significant (*BF*_ROPE_ = 0.014).

### Readability analysis

Using the FORCAST algorithm, we calculated the equivalent grade level of the full TAS-20 (including instructions) to be 10.2 (i.e., appropriate for individuals at the reading level of an American 10^th^-grader [chronological age 15–16 years] after the second month of class). This estimate was several grades higher than that produced using the Flesch–Kincaid algorithm (FKGL = 6.7; FRE = 73: “Fairly Easy”). Using the FORCAST algorithm, the eight items contributing to the GAFS-8 demonstrated a grade level of 8.8, indicating a moderate decrease in word difficulty compared to the full scale. This decreased reading level compared to the TAS-20 was also reflected in the Flesch–Kincaid measures (FKGL = 4.5; FRE = 86: “Easy”). Thus, in addition to improving the psychometric properties of the measure, our item reduction procedure appeared to remove some of the more difficult-to-read items of the TAS-20.

## Discussion

While alexithymia is theorized to account for many traits associated with the autism phenotype [40–52], studies to date have not typically assessed the psychometric properties of alexithymia measures in the autistic population, and the suitability of most alexithymia measures for comparing autistic and non-autistic individuals in an unbiased manner remains largely unknown. In the current study, we performed a rigorous examination of the psychometric properties of the TAS-20, the most widely used measure of self-reported alexithymia, in a large and diverse sample of autistic adults. Overall, we found the TAS-20 questionnaire to have a number of psychometric issues when completed by autistic adults, including a poorly fitting measurement model, several items that are minimally related to the overall alexithymia construct, and items that function differentially when answered by autistic and non-autistic respondents.

In response to these issues, we performed an empirically based item reduction of the TAS-20 questionnaire, which resulted in the identification of eight items that were strong indicators of the TAS-20’s “general alexithymia” factor and also answered in an equivalent manner by autistic and non-autistic participants. The factor score calculated from these items (the GAFS-8) was found to be psychometrically robust in both general population and autistic samples, displaying strong model-data fit to a unidimensional structure, high score reliability, strong nomological validity, and practically ignorable amounts of I-DIF between diagnostic groups and subgroups of autistic and general population adults. Readability analysis also indicated that the eight GAFS-8 items had a lower average reading level than the full TAS-20, indicating that this novel score may be particularly useful for younger, less educated, or less cognitively able respondents. In sum, our findings suggest that the GAFS-8 is a reliable and valid measure of alexithymia suitable for use by autistic adults as well as adults in the general population, although its improved ability to measure alexithymia over the full TAS-20 has only so far been demonstrated for adults on the autism spectrum. Note that the GAFS-8 does not introduce a new instrument but rather is a novel score that can be calculated from TAS-20 item scores; its title does not reference the TAS-20 due to copyright of that instrument.

While the 20-item TAS possessed adequate composite score reliability in our sample, bifactor confirmatory factor models failed to support the theorized structure of the questionnaire in the autistic population. The TAS-20 items assessing the EOT facet of the alexithymia construct and the form’s reverse-coded items were particularly problematic, both exhibiting poor subscale reliabilities and contributing little common variance to the general alexithymia factor. These psychometric issues were further confirmed in our general population HPP sample, indicating that these problems were not unique to the autistic population. However, as the HPP sample did not complete all 20 TAS-20 items, the observed misfit of this model in the general population should be considered preliminary and warrants replication. Nevertheless, removal of the EOT and reverse-coded items from the model greatly improved overall fit in both samples, but three additional items needed to be removed in order to meet our a priori standards of adequate IRT model fit and negligible I-DIF by diagnostic group. The final item set used to calculate the GAFS-8 consisted of five DIF items (1, 6, 9, 13, and 14) and three DDF items (2, 11, and 12) that ostensibly form the core of the “general alexithymia” construct measured by the TAS-20 total score. Using item response theory, we generated norm-referenced GAFS-8 scores that are immediately interpretable on the scale of a Z-score (i.e., *M* = 0, SD = 1) and can similarly be scaled to the familiar T-score metric (*M* = 50, SD = 10). As GAFS-8 scores are both norm-referenced and psychometrically robust, we believe they present a viable alternative to TAS-20 total scores in any study protocol that includes the TAS-20 or a subset of TAS-20 items. To facilitate the calculation and use of the GAFS-8 latent trait scores in alexithymia research, we have created an easy-to-use online scoring tool (available at http://asdmeasures.shinyapps.io/alexithymia) that converts TAS-20 item responses into general population-normed GAFS-8 scores and corresponding T-scores.

In addition to deriving a psychometrically robust general alexithymia score from the TAS-20, the current study also sheds light on the areas of the form that are most psychometrically problematic, notably the EOT subscale. This subscale was the primary driver of poor TAS-20 model fit in the current study, and even when method factors were appropriately modeled, the reliability of the EOT subscale score was unacceptably low. Notably, it is not uncommon for researchers to perform subscale-level analyses using the TAS-20, examining correlations between DIF/DDF/EOT subscale scores and other constructs of theoretical interest [3, 61]. As the EOT scale of the TAS-20 does not appear to measure a single coherent construct (or alexithymia itself, in the current samples), we strongly question the validity of analyses conducted using this subscale by itself in autistic adults and recommend that autism researchers restrict their use of the TAS-20 to only the total score and potentially the DIF/DDF subscales.

Tests of convergent and divergent validity of the GAFS-8 score were largely in line with prior studies using the TAS-20, indicating that self-reported alexithymia is moderately to strongly correlated with autistic traits, repetitive behaviors, internalizing psychopathology, suicidality, and poorer quality of life. These correlations were approximately equivalent in magnitude to those calculated using the TAS-20 total score (though slightly stronger in most cases), indicating that removal of EOT and reverse-coded items from the TAS-20 does not meaningfully affect the nomological validity of the resulting alexithymia score in autistic individuals. Again, this finding warrants replication in non-autistic samples with complete TAS-20 data. Relationships were also observed between GAFS-8 scores and sex, age of autism diagnosis, and education level, although these effects were small enough to be practically insignificant (i.e., |*r*|s < 0.2 and |*d*|s < 0.2). Moreover, despite a fairly large correlation between GAFS-8 scores and neuroticism, partial correlation analyses demonstrated that alexithymia still explained substantial unique variance in autism symptomatology, depression, and generalized anxiety over and above that accounted for by neuroticism. However, partial correlations with somatic symptom burden, social anxiety, and suicidal ideation failed to exceed the pre-specified interval null hypothesis, indicating that alexithymia in the autistic population only predicts these symptom domains insofar as it correlates positively with trait neuroticism. A particularly important future direction in alexithymia research will be to re-examine studies wherein alexithymia was found to be a “more useful predictor” of some clinical outcome when compared to autistic traits [61]; to date, these studies have not taken trait neuroticism in account, and we believe that it is quite likely that alexithymia may no longer be a stronger predictor of many other constructs once variance attributable to neuroticism is partialed out. Moreover, as alternative measures of alexithymia such as the TSIA [76], BVAQ, and Perth Alexithymia Questionnaire (PAQ) [75] do not correlate highly with neuroticism [70, 77, 78], future research should also investigate the degree to which alexithymia measured multimodally continues to predict internalizing psychopathology in the autistic population and other clinical groups of interest.

One particularly surprising finding is the poor correlation between alexithymia and somatic symptom burden, given the theoretical status of alexithymia as a potential driver of somatization and a large literature showing relationships between these constructs [3]. One particular reason that this correlation may be substantially attenuated is that the GAFS-8 did not include TAS-20 item 3 (describing an individual having “physical sensations that even doctors don’t understand”) due to substantial I-DIF across diagnostic groups. In addition to assessing the experience of undifferentiated emotions common in alexithymia, TAS-20 item 3 also seemingly captures the phenomenon of medically unexplained symptoms. We confirmed that this was in fact the case in our SPARK sample, as the polyserial correlation between this item and PHQ-15 total scores was very high (*r*_poly_ = 0.492 [0.435, 0.543]) and very minimally attenuated after controlling for overall alexithymia as measured by the GAFS-8 latent trait score (*r*_p,poly_ = 0.424 [0.364, 0.485], *BF*_ROPE_ = 4.79 × 10^10^). Notably, a recent study has found that item 3 of the TAS-20 is the single most important item when discriminating individuals with a functional somatic condition (fibromyalgia) from healthy controls [166], providing additional evidence to support our suspicion that this particular item drives much of the correlation between the TAS-20 and somatic symptomatology. Additional work in this area should attempt to measure alexithymia in a multimodal manner (e.g., simultaneously administering the GAFS-8, a second self-report questionnaire such as the BVAQ [64] or PAQ [75], an observer-report measure such as the Observer Alexithymia Scale [167] or TAS-20 informant report [168], and an interview measure such as the TSIA), as such multi-method studies are able to separate out the degree of variance in these measures due to alexithymia versus construct-irrelevant method factors (such as self-report questionnaire response styles). Multi-method alexithymia work is almost entirely absent from the autism literature [169], although such work on a larger scale (i.e., with samples large enough to fit latent variable models) is necessary to determine which relationships between alexithymia and important covariates of interest (e.g., somatization, neuroticism, autism symptoms, emotion recognition, and psychopathology) are due to the latent alexithymia construct or measurement artifacts specific to certain alexithymia assessments or response modalities.

This work has meaningful implications for the study of alexithymia in the autistic population and in general, as it strongly supports the use of the GAFS-8 as a general-purpose measure of alexithymia in autistic adults and provides preliminary evidence of its utility in the general adult population. These findings are particularly useful for autism research, as they indicate that the GAFS-8 can be used to compare levels of alexithymia between autistic and general population samples without worry that differences in scores are significantly biased by qualitative differences in the ways individuals in each group answer the questionnaire items. Moreover, the between-group difference in GAFS-8 scores (*d* = 1.014) was approximately 15% larger than the same group difference in TAS-20 scores (*d* = 0.880), indicating that the GAFS-8 may be better able to discriminate between autistic and non-autistic adults than the TAS-20 total score. Although the current study did not validate this novel score for use in other clinical populations where alexithymia is a trait of interest (e.g., individuals with eating disorders, functional somatic syndromes, substance abuse disorders, or general medical conditions), future studies in these populations are warranted to determine whether the GAFS-8 remains a robust measure of general alexithymia in those groups as well.

## Limitations

This study has a number of strengths, including its large and diverse sample of both autistic and non-autistic participants, robust statistical methodology, wide array of clinical measures with which to assess the validity of the GAFS-8, and consideration of the role that neuroticism plays in explaining relationships between alexithymia and internalizing psychopathology. However, this investigation is not without limitations. Most notably, the two samples of participants (from SPARK and HPP, respectively), while both recruited online, were drawn from different studies with dissimilar protocols and different numbers of TAS-20 items administered. The HPP sample completed a version of the TAS-20 questionnaire with four items omitted. Thus, in order to estimate TAS-20 total scores in this group of individuals, we were required to impute those items for all 721 participants with an unknown degree of error. This situation particularly limits the degree to which we can draw inferences about the adequacy of the full TAS-20 in the general population. Interestingly, the HPP sample reported TAS-20 scores that were 1.5–6 points larger on average than previous large-scale general population studies using the TAS-20 [19, 170], and it is thus unclear whether the imputation of four items using data from an autistic sample artificially inflated these scores. However, as the GAFS-8 was not calculated using any of the imputed items, we can be reasonably confident that the scores on this measure genuinely reflect individual differences in the underlying alexithymia construct in the current general population sample. Moreover, supplemental analyses using only the 16 completed items in both groups were nearly identical to those conducted using the imputed scores, further supporting the validity of our conclusions. Nevertheless, additional research that compares the psychometric properties of the GAFS-8 to the full TAS-20 in the general population is needed in order to support our preliminary findings.

An additional limitation is that the HPP sample was not screened for autism diagnoses, and there remains a possibility that some of these individuals could have met diagnostic criteria for autism or had a first-degree relative on the autism spectrum. However, previous studies have indicated that a small portion of autistic individuals (i.e., approximately 2% per current prevalence estimates [95]) in an otherwise neurotypical sample is insufficient to substantially bias parameter estimates or attenuate differential item functioning [83], leading us to believe that the current group comparisons remain valid. Nevertheless, the HPP sample was only assessed on a small number of relevant demographic domains, leaving unanswered questions about the relationships between alexithymia as measured by the GAFS-8 and many demographic and clinical variables of interest in general population adults. Individuals in the HPP sample also did not complete measures of psychopathology or neuroticism, which may account for a substantial portion of the diagnostic group difference in alexithymia scores. Fortunately, as the GAFS-8 score can be calculated from item-level TAS-20 data, many extant datasets currently exist that can provide answers to these questions, further supporting or refuting the validity of the GAFS-8 as a measure of alexithymia in the general population and other groups of interest.

In addition to the limitations of the HPP sample, several limitations of the better-characterized SPARK sample were also present. As discussed in our previous work with this sample [83, 88, 97, 98], it is not representative of the autistic population, having a higher proportion of females, a higher average education level, later mean age of autism diagnosis, and a higher prevalence of co-occurring anxiety and depressive disorders than is expected in this population [171]. The sex ratio of this sample is particularly divergent from that seen in most clinical samples (i.e., 3–4:1 male-to-female ratio [172]), and thus, the over-representation of females may affect group-level parameters such as the mean alexithymia score modeled for the autistic population in this sample. Nevertheless, a strength of the IRT method is the fact that unrepresentative samples are able to still provide unbiased item parameter estimates provided that there is minimal I-DIF between subgroups of the population of interest [173]. As we found little meaningful I-DIF within autistic adults across numerous demographic and clinical groupings, we feel very confident that the parameter estimates generated from the current study will generalize well to future samples that are demographically dissimilar. In addition, as SPARK does not include data on cognitive functioning, we were unable to determine whether the GAFS-8 demonstrated relationships with verbal IQ, as has been previously reported with TAS-20 scores in the autistic population [52]. It remains unclear whether this relationship is an artifact of the generally high reading level of the TAS-20 (which would ideally be attenuated using just the GAFS-8 score) or a manifestation of some other relationship between alexithymia and verbal intelligence (e.g., language impairment [reflected in reduced verbal intelligence] is a developmental precursor of alexithymia, as posited by the recently proposed “alexithymia-language hypothesis” [174]). Future studies of alexithymia in the autistic population should incorporate measures of verbal and nonverbal cognitive performance, examining the relationships between these constructs and alexithymia and additionally testing whether self-report measures such as the GAFS-8 function equivalently in autistic adults with higher and lower verbal abilities.

Another limitation concerns the correspondence of the GAFS-8 to the theoretical alexithymia construct itself, as initially proposed by Sifneos and colleagues [2, 175]. As noted previously, alexithymia is made up of four interrelated facets: DIF, DDF, EOT, and difficulty fantasizing (DFAN), the latter two of which are not measured directly by the GAFS-8. Because of this, the questionnaire arguably lacks content validity compared to the full TAS-20 or four-dimensional measures such as the TSIA. However, our results indicated that the EOT factor measured by the TAS was not highly correlated with the “general alexithymia” factor (which had its highest loadings on DIF/DDF items; see also [176]) and therefore does not adequately measure this facet of the alexithymia construct. Other measures, such as the PAQ [75], have found that a more restricted EOT factor (primarily reflecting one’s tendency to not focus attention on one’s own emotions) correlates much more highly with other measures of the alexithymia construct, likely representing a better operationalization of the EOT facet of alexithymia. In addition, items reflecting the DFAN dimension of alexithymia have displayed poor psychometric properties in both questionnaire and interview measures, and there is currently debate as to whether these items truly measure part of the alexithymia construct [3, 34, 177–180]. Moreover, studies in the autism population examining the correlates of alexithymia have found the DIF and DDF subscales to be most important in predicting clinically meaningful outcomes such as depression, anxiety, and social communication difficulties [60]. Thus, it is our belief that the “core” of alexithymia (consisting of difficulty identifying and describing emotional experiences) is likely sufficient to represent this construct in autistic adults, particularly when options to measure the EOT and DFAN facets are psychometrically inadequate. Although there is ongoing debate over whether the definition of alexithymia should be changed to exclude certain historically relevant facets of the construct [175, 180], we believe that construct definitions *should* change over time, incorporating relevant findings such as empirical tests of latent variable models. Future research in alexithymia would greatly benefit from additional psychometric studies that aim to generate optimal instruments to measure all facets of the alexithymia construct, coupled with tests of the incremental validity of the EOT/DFAN trait facets over and above a score composed of solely DIF/DDF items, such as the GAFS-8.

A final limitation of our study is the fact that we were unable to test all meaningful psychometric properties of the GAFS-8. In particular, our study was cross-sectional, necessarily prohibiting us from assessing test–retest reliability, temporal stability, and I-DIF across repeated test administrations. Additionally, as alexithymia appears to be amenable to change with psychological interventions [181, 182], future studies should also investigate whether the GAFS-8 latent trait score is sensitive to change, and if so, determine the minimal clinically important difference in this score. Additional psychometric characteristics that could be tested include convergent validity with other alexithymia measures such as the PAQ or TSIA, predictive validity for clinically meaningful outcomes such as response to psychotherapy, and I-DIF across language, culture, medium of administration (e.g., pen and paper vs. electronic), age group (e.g., adolescents vs. adults), and other diagnostic contrasts beyond the autism population. As inferences in the psychological science are only as reliable and valid as the measures they utilize [183], we encourage autism researchers and individuals in psychological science more broadly to consider the importance of measurement in their science and to devote more effort to investigating and justifying the ways in which complex psychological constructs such as alexithymia are operationalized.

## Conclusions

The TAS-20 is a widely used measure of alexithymia that has more recently become the de facto measure of choice for this construct in the autism literature. However, this measure has so far lacked robust psychometric evidence for its reliability and validity in the population of autistic adults. Leveraging two large datasets of autistic and general population adults, we performed an in-depth investigation of the TAS-20 and its measurement properties in autistic adults, revealing several psychometric shortcomings of this commonly used questionnaire. Using empirically driven item reduction, we were able to identify a unidimensional set of TAS-20 items that could be used to assess general alexithymia equivalently in samples of both autistic and non-autistic adults (the GAFS-8 score). Furthermore, in order to allow others to utilize the population-normed latent trait scores generated by our IRT model, we have created a user-friendly online score calculator for the GAFS-8 (https://asdmeasures.shinyapps.io/alexithymia) that is freely available to interested researchers who wish to calculate this score from existing TAS-20 data. Although the measurement properties of the GAFS-8 were strong in this study, we stress that this single measure should not be considered the “gold standard” of alexithymia measurement in autism or any other population. In agreement with the original authors of the TAS-20 [3], we recommend that researchers interested in robustly measuring the alexithymia construct use multiple measures that include both self- and proxy-report questionnaires, ideally supplemented by observational or interview measures. Additional studies are still needed to fully explore the psychometric properties of the GAFS-8 and replicate its utility as a measure of general alexithymia in other samples (particularly general population adults and non-autistic clinical populations), but in light of the current study, we believe that this empirically derived score has potential to improve the measurement of alexithymia both within and outside the field of autism research.

## Supplementary Information

**Additional file 1.** Supplementary Methods and Tables.

## Data Availability

Approved researchers can obtain the SPARK population dataset described in this study by applying at https://base.sfari.org. Open data from the Human Penguin Project can be downloaded from Open Science Framework (https://osf.io/2rm5b/). Custom R code to perform the analyses in this paper can be found on the ResearchGate profile of the corresponding author (https://www.researchgate.net/profile/Zachary-Williams-6/publications). The remainder of research materials can be obtained from the corresponding author upon request.

## Abbreviations

ADHD: Attention deficit hyperactivity disorder
BDI-II: Beck Depression Inventory-II
BVAQ: Bermond–Vorst Alexithymia Questionnaire
BFNE-S: Brief Fear of Negative Evaluation—Short
cML: Categorical maximum likelihood
CFA: Confirmatory factor analysis
CFI: Comparative fit index
CrI: Credible interval
I-DIF: Differential item functioning
DDF: Difficulty describing feelings
DFAN: Difficulty fantasizing
DIF: Difficulty identifying feelings
GAFS-8: Eight-item general alexithymia factor score
ESSD: Expected score standardized difference
ETSSD: Expected test score standardized difference
ECV: Explained common variance
EOT: Externally oriented thinking
FDR: False discovery rate
FKGL: Flesch–Kincaid grade level
FRE: Flesch Reading Ease
WHOQOL-4: Four-Item World Health Organization Quality of Life Score
GAD-7: Generalized Anxiety Disorder-7
HPP: Human Penguin Project
I-ECV: Item explained common variance
IRT: Item response theory
MAP: Maximum a posteriori
PHQ-15: Patient Health Questionnaire-15
PUC: Percentage of uncontaminated correlations
PAQ: Perth Alexithymia Questionnaire
RBS-R: Repetitive Behavior Scale—Revised
RS: Ritualistic/sameness
RMSEA: Root mean square error of approximation
SM: Sensory motor
SPARK: Simons Powering Autism Research Knowledge cohort
SRS-2: Social Responsiveness Scale—Second Edition
SRMR: Standardized root mean square residual
TAS: Toronto Alexithymia Scale
TSIA: Toronto Structured Interview for Alexithymia
TLI: Tucker–Lewis Index
UETSDS: Unsigned expected test score difference in the sample
wABC: Weighted area between curves
