# Supplementary material for: Improving the measurement of alexithymia in autistic adults: a psychometric investigation of the 20-item Toronto Alexithymia Scale and generation of a general alexithymia factor score using item response theory
Source: Mol Autism. 2021 Aug 10;12:56. doi: 10.1186/s13229-021-00463-5 (PMC8353782; doi:10.1186/s13229-021-00463-5)
Supplement: Supplementary file 1 — Additional file 1. Supplementary Methods and Tables. [file 13229_2021_463_MOESM1_ESM.docx]

**Supplemental Material:**

**Improving the Measurement of Alexithymia in Autistic Adults:**

**A Psychometric Investigation of the Twenty-item Toronto Alexithymia Scale and**

**Generation of a General Alexithymia Factor Score Using Item Response Theory**

Zachary J. Williams & Katherine O. Gotham

Table of Contents

[Supplemental Methods S2](#_Toc78199677)

[Questionnaire Measures S2](#_Toc78199678)

[***Social Responsiveness Scale–Second Edition (SRS-2)*** S2](#_Toc78199679)

[***Repetitive Behavior Scale–Revised, Self-Report (RBS-R)*** S2](#_Toc78199680)

[***Beck Depression Inventory–II (BDI-II)*** S3](#_Toc78199681)

[***Generalized Anxiety Disorder–7 (GAD-7)*** S4](#_Toc78199682)

[***Brief Fear of Negative Evaluation Scale–Short (BFNE-S)*** S4](#_Toc78199683)

[***Modified Patient Health Questionnaire–15 (PHQ-15)*** S5](#_Toc78199684)

[***International Personality Item Pool 10-item Neuroticism Scale (IPIP-N10)*** S5](#_Toc78199685)

[***Four Item World Health Organization Quality of Life Score (WHOQOL-4)*** S6](#_Toc78199686)

[Bayesian Model Estimation S6](#_Toc78199687)

[***Linear and Polyserial Correlations*** S6](#_Toc78199688)

[***Bayesian Unequal-variance t-tests*** S8](#_Toc78199689)

[Supplemental Table S1 S9](#_Toc78199690)

[Supplemental Table S2 S10](#_Toc78199691)

[Supplemental References S11](#_Toc78199692)

# **Supplemental Methods**

## **Questionnaire Measures**

### ***Social Responsiveness Scale–Second Edition (SRS-2)***

The SRS-2 Adult Self Report (Bruni, 2014; Constantino & Gruber, 2012) is a widely used 65-item measure of quantitative autistic traits designed for use both in samples of autistic and general population adults. Items are scored on a 4-point Likert scale from *Not true* to *Almost always true*, with higher scores indicating higher levels of autistic symptomatology. T-scores (*M* = 50, *SD* = 10) are also available for individuals based on sex and the specific form used. In the current study, the SRS-2 adult self-report form was used in the SPARK cohort as a measure of quantitative autistic traits, from which overall T-scores were derived. Model-based coefficients of reliability and general factor saturation in the SPARK sample were excellent (ω_T_ = 0.955, ω_H_ = 0.920) when derived from a bifactor model with a common method factor for the reverse-coded items.

### ***Repetitive Behavior Scale–Revised, Self-Report (RBS-R)***

The RBS-R (Bodfish et al., 2000; Lam & Aman, 2007; McDermott et al., 2020) is a 43-item measure of repetitive behaviors associated with autism, originally designed for caregivers to report on the behaviors of their children. Items are scored on a four-point Likert scale from *Behavior does not occur* to *Behavior occurs and is a severe problem*. When deciding on a score for each item, respondents are directed to how frequently the behavior occurs, how difficult the behavior is to interrupt, and how much the behavior interferes with ongoing events, resulting in a global severity rating for each listed behavior. Recently, this form has been adapted for self-report by cognitively-able adolescents and adults on the autism spectrum, and preliminary psychometric work has supported the reliability and convergent validity of self-reported *Sensory Motor* (SM; 7 items) and *Ritualistic/Sameness* (RS; 11 items) subscale scores (Bishop et al., 2013) as indicators of “lower-order” and “higher-order” repetitive behaviors, respectively (McDermott et al., 2020). Although the RBS-R contains additional subscales, we constrained our analyses to these two scores given that the other scores have not been validated in the self-report format. In the SPARK sample, model-based reliability coefficients based on a two-factor oblique solution were adequate for both the self-reported SM and RS subscales (ω_S_ = 0.811 and 0.903, respectively).

### ***Beck Depression Inventory–II (BDI-II)***

The BDI-II (Beck et al., 1996) is a widely used 21-item self-report measure of depressive symptoms experienced over the past two weeks. The severity of each symptom is rated on a four-point Likert scale with unique anchors for each question. This measure is typically modeled as a bifactor structure, with two specific factors representing “cognitive-affective” and “somatic-vegetative” symptoms of depression, although almost all reliable variance is attributable to the general depression factor (Brouwer et al., 2013; Williams et al., 2021). The BDI-II is a reliable and valid measure of depression symptom severity in the autistic adult population, and normed latent trait scores on the general depression factor have been derived specifically to quantify general depression symptomatology in autistic adults (Williams et al., 2021). Using the online BDI-II scoring tool developed previously by our research group (<https://asdmeasures.shinyapps.io/bdi_score/>), we calculated autism-specific latent depression scores for this measure to be used in the current study. These scores exhibited excellent reliability in our SPARK sample (median *r*_xx_ = 0.915, inter-quartile range [0.839, 0.970]). In addition to the BDI-II latent trait score, we also extracted item-level scores on BDI-II item 9 (*Suicidal Thoughts or Wishes*), which we used to measure the current degree of suicidality.

### ***Generalized Anxiety Disorder–7 (GAD-7)***

The GAD-7 (Spitzer et al., 2006) is a self-report measure of generalized anxiety disorder experienced over the previous two weeks. Items are rated on a four-point Likert scale ranging from *not at all* to *nearly every day*. Scores range from 0 to 21, with scores of 10 or greater indicating clinically significant anxiety. This measure has not been formally validated in autistic adults, although prior studies have confirmed hypothesized relationships with related constructs in the autistic population, supporting its validity in the current sample (Hull et al., 2019; Russell et al., 2020; Williams et al., 2021). In our SPARK sample, the GAD-7 total score exhibited strong reliability based on a unidimensional model (ω = 0.912).

### ***Brief Fear of Negative Evaluation Scale–Short (BFNE-S)***

The BFNE (Leary, 1983) is a 12-item short-form of the 30-item Fear of Negative Evaluation Scale (Watson & Friend, 1969) and a commonly used self-report measure of social anxiety. Items are rated on a 5-point Likert scale from *not at all* to *extremely*. A commonly variant, the BFNE-S (Carleton et al., 2011), eliminates the four reverse-coded items, improving the psychometric properties of the overall measure. The BFNE and its variations are frequently used to measure social anxiety in autistic adolescents and adults (Capriola et al., 2017; Maddox & White, 2015; e.g., South et al., 2017; Spain et al., 2016), and preliminary psychometric work has supported the reliability and validity of both the BFNE and BFNE-S in this population (Boulton & Guastella, 2020). The full BFNE was administered as part of the current study, and from this form, we calculated scores on the eight-item BFNE-S. In the current SPARK sample, reliability of the BFNE-S composite score was excellent based on a unidimensional model (ω = 0.951).

### ***Modified Patient Health Questionnaire–15 (PHQ-15)***

The PHQ-15 (Kroenke et al., 2002) is a self-administered screening measure assessing the impact of 15 commonly-reported somatic symptoms (e.g., joint pain, dizziness, fatigue) over the past four weeks. Each item is rated on a three-point Likert scale from *Not bothered at all* to *Bothered a lot*. The PHQ-15 has been extensively used in medical, psychiatric, and general population samples (Kroenke et al., 2010). Although this measure is used routinely in psychiatric research, we are unaware of any study that has previously utilized it in a sample of autistic adults. The current study used a slightly modified version of the PHQ-15 that omitted the item assessing pain or problems during sexual intercourse and assessed the burden of somatic symptoms over a three-month time period. The female-specific item on menstrual cramps was also excluded from all analyses to allow for scores to be based on the same items for individuals of all genders. Using a bifactor model with specific factors for cardiopulmonary symptoms and gastrointestinal symptoms (based on (Witthöft et al., 2016)), this modified form of the PHQ-15 displayed adequate model-based reliability and general factor saturation in the current SPARK sample (ω_T_ = 0.872, ω_H_ = 0.774).

### ***International Personality Item Pool 10-item Neuroticism Scale (IPIP-N10)***

Ten items from the international personality item pool (Goldberg et al., 2006), originally from the Multidimensional Personality Questionnaire’s “Stress Reaction” subscale (Tellegen & Waller, 2008) were utilized to form a measure of neuroticism (IPIP-N10) for use in this study. Items were rated on a five-point Likert scale from *Strongly Disagree* to *Strongly Agree*, and half of the items were reverse-coded. In the current SPARK sample, model-based coefficients of reliability and general factor saturation were found to be acceptable (ω_T_ = 0.891, ω_H_ = 0.838) when derived from a bifactor model with a common method factor for the reverse-coded items.

### ***Four Item World Health Organization Quality of Life Score (WHOQOL-4)***

The WHOQOL-4 is a four-item measure of self-reported quality of life (QoL) derived from the 26-item WHOQOL-BREF, a widely-used quality of life measure that has previously been validated in autistic adults (McConachie et al., 2018). Items are rated on a five-point Likert scale with varying response options. The WHOQOL-4 index contains the following WHOQOL-BREF items: 1 (*How would you rate your quality of life?*), 5 (*How much do you enjoy life?*), 6 (*To what extent do you feel your life to be meaningful?*), and 19 (*How satisfied are you with yourself?*). The composite score derived from these items has been validated in a sample of autistic adults that overlaps substantially with the current sample (Williams & Gotham, 2021). In that study, the WHOQOL-4 demonstrated excellent fit to a unidimensional model, no meaningful local dependence, no differential item functioning across demographic and clinical groups, and high marginal reliability (ρ_xx_ = 0.890). The measure also demonstrated strong convergent validity with the Autism Spectrum Quality of Life Score (ASQoL; McConachie et al., 2018) a measure of autism-relevant quality of life (*r* = 0.604). WHOQOL-4 scores are calculated as the mean of all four items and range from 1 (“Very Poor”) to 5 (“Very Good”).

## **Bayesian Model Estimation**

### ***Linear and Polyserial Correlations***

Robust bivariate correlations between eight-item general alexithymia factor scores (GAFS-8 scores) and continuous variables were estimated using an intercept-only multivariate Student-*t* regression model, with the parameter of interest being the residual correlation coefficient (Kurz, 2019). This model was estimated in Stan using the *brms* R package (Bürkner, 2017) with weakly informative priors, including default scaled Student­-*t*_3_ priors on parameters μ and log(σ), a Gamma(2, 0.1) prior on ν (degrees of freedom for the *t*-distribution), and a Lerandowski-Kurowicka-Joe (LKJ) prior on the residual correlation coefficient (Lewandowski et al., 2009), with parameter η = 2 to reduce the prior probability of extreme correlations. Robust partial correlations were calculated in a similar manner as bivariate correlations, except a trivariate *t*-distribution was estimated, producing a 3 x 3 correlation matrix for all variables (i.e., alexithymia scores, scores on the correlate of interest, and neuroticism scores). We then calculated *r*_xy,z_ from *r*_xy_, *r*_xz_, and *r*_yz­_ for each posterior sample using the standard estimator based on bivariate correlations (see Wetzels & Wagenmakers, 2012 for additional details), allowing us to estimate the posterior distribution of the partial correlation coefficient *r*_p_. Model parameters were estimated via Markov chain Monte Carlo (MCMC) using the No U-turn Sampler (Homan & Gelman, 2014), with posterior distributions of each parameter estimated using 20,000 post-warmup MCMC draws from five Markov chains. Parameter summaries from these posterior distributions were operationalized as the posterior median and the 95% highest-density credible interval (CrI). Convergence for each model was confirmed by examination of Markov chain trace plots, as well as values of the Gelman–Rubin (1992) convergence diagnostic < 1.01.

Bivariate polyserial correlations and partial polyserial correlations between GAFS-8 scores and ordinal variables (e.g., the BDI-II suicidality item) were estimated using a Bayesian ordered-probit regression model, with correlation coefficients calculated from the raw regression parameters and the standard deviation of the predictor variable (residualized on the covariate in the case of partial correlations) according to the methods described by Breen et al. (2014). The scale of the latent outcome variable was fixed to a standard normal distribution, and weakly-informative prior distributions were placed on all other model parameters, including a Student *t*_3_(0, 2.5) prior on intercept terms and Normal(0, 1) prior on all unstandardized slope parameters. Model estimation procedures and convergence checks were identical to those described above for the linear correlation coefficients.

### ***Bayesian Unequal-variance t-tests***

When comparing GAFS-8 latent trait scores between categorical groups (e.g., men and women), we utilized a Bayesian *t*-test similar to the BEST procedure proposed by Kruschke (2013). However, a notable difference is that in our model, priors were not dependent on the data, dependent variables were standardized (*M* = 0, *SD* = 1). The standardized outcome was fit to an unequal-variances *t*-test model in *brms*, with a Normal(0, 1) prior on regression coefficients (i.e., the intercept term, mean difference between groups, and mean difference in log(σ) between groups), a Normal(0, 1) prior on log-transformed standard deviation parameters for each group, and a Gamma(2, 0.1) prior on ν, the degrees of freedom of the *t-*distribution. Posterior distributions of the parameters were based on 40,000 post-warmup MCMC draws from five Markov chains. The primary parameter of interest was the standardized mean difference between groups (i.e., Cohen’s *d*, calculated as the difference in means divided by the square root of the pooled variance), which we summarized using the posterior median and 95% CrI.

# **Supplemental Table S1**

***TAS-20 Factor Loadings for Confirmatory Bifactor Model in Autistic Adults (N = 743)***

| **TAS-20 Item Number** | **λ_G_** | **λ_DIF_** | **λ_DDF_** | **λ_EOT_** | **λ_REV_** | ***h^2^*** | ***I-ECV*** |
| --- | --- | --- | --- | --- | --- | --- | --- |
| 1 | 0.841 | 0.174 | — | — | — | 0.737 | 0.959 |
| 2 | 0.821 | — | 0.302 | — | — | 0.765 | 0.881 |
| 3 | 0.420 | 0.635 | — | — | — | 0.580 | 0.304 |
| 4 | 0.627 | — | 0.674 | — | 0.311 | 0.944 | 0.416 |
| 5 | **-0.116** | — | — | 0.341 | 0.267 | 0.201 | 0.067 |
| 6 | 0.827 | 0.152 | — | — | — | 0.706 | 0.967 |
| 7 | 0.595 | 0.725 | — | — | — | 0.880 | 0.402 |
| 8 | **0.172** | — | — | 0.659 | — | 0.464 | 0.063 |
| 9 | 0.798 | 0.288 | — | — | — | 0.720 | 0.885 |
| 10 | **0.264** | — | — | 0.214 | 0.448 | 0.316 | 0.221 |
| 11 | 0.749 | — | 0.166 | — | — | 0.588 | 0.953 |
| 12 | 0.661 | — | 0.147 | — | — | 0.458 | 0.953 |
| 13 | 0.714 | 0.252 | — | — | — | 0.574 | 0.889 |
| 14 | 0.677 | 0.172 | — | — | — | 0.488 | 0.939 |
| 15 | 0.550 | — | — | 0.211 | — | 0.347 | 0.872 |
| 16 | **0.202** | — | — | 0.262 | — | 0.109 | 0.373 |
| 17 | 0.622 | — | 0.220 | 0.000 | — | 0.435 | 0.889 |
| 18 | **0.105** | — | — | 0.087 | 0.388 | 0.170 | 0.065 |
| 19 | **0.222** | — | — | 0.316 | 0.775 | 0.750 | 0.066 |
| 20 | **0.311** | — | — | 0.231 | — | 0.150 | 0.643 |

*Note.* General factor loadings less than 0.4 are highlighted in bold. λ_G_ = general factor loading; λ_DIF_ = difficulty identifying feelings factor loading; λ_DDF_ = difficulty describing feelings factor loading; λ_EOT_ = externally-oriented thinking factor loading; λ_REV_ = reverse-coded item method factor loading; *h*^2^ = communality (sum of squared loadings); *I-ECV* = item explained common variance.

# **Supplemental Table S2**

***Zero-order Correlations Between TAS-20 Total Scores and Other Clinical Measures in SPARK Sample***

| **Covariate** | **TAS-20 Correlation [95% CrI]** | ***BF*_ROPE_** | $\boldsymbol{P}\mathbf{(ROPE}\boldsymbol{\vert}\mathbf{Data}$**)** | **GAFS-8 Correlation [95% CrI]** | **∆*r*** |
| --- | --- | --- | --- | --- | --- |
| SRS-2 | 0.649 [0.606, 0.691] | **1.08 × 10^25^** | <0.001 | 0.642 [0.598, 0.686] | 0.007 |
| RBS-R SM | 0.363 [0.297, 0.425] | **5.01 × 10^4^** | <0.001 | 0.385 [0.322, 0.444] | -0.022 |
| RBS-R RS | 0.406 [0.345, 0.468] | **1.69 × 10^7^** | <0.001 | 0.432 [0.372, 0.494] | -0.026 |
| BDI-II | 0.391 [0.329, 0.454] | **1.96 × 10^6^** | <0.001 | 0.420 [0.358, 0.480] | -0.029 |
| GAD-7 | 0.387 [0.324, 0.448] | **3.77 × 10^5^** | <0.001 | 0.423 [0.360, 0.481] | -0.036 |
| BFNE-S | 0.292 [0.226, 0.361] | **94.0** | 0.005 | 0.358 [0.292, 0.423] | -0.066 |
| PHQ-15 | 0.286 [0.219, 0.355] | **53.3** | 0.008 | 0.275 [0.208, 0.346] | 0.011 |
| WHOQOL-4 | -0.354 [-0.417, -0.289] | **3.30 × 10^5^** | <0.001 | -0.357 [-0.419, -0.291] | 0.003 |
| Suicidality | 0.260 [0.178, 0.341] | 2.32 | 0.078 | 0.303 [0.222, 0.382] | -0.043 |
| IPIP-N10 | 0.433 [0.373, 0.492] | **1.81 × 10^7^** | <0.001 | 0.475 [0.416, 0.531] | -0.042 |

*Note.* Correlations are presented between each covariate and the total score of the 20-item Toronto Alexithymia Scale (TAS-20) as well as the eight-item general alexithymia factor score (GAFS-8). The ∆*r* column reports the median correlation difference (TAS-20 – GAFS-8). *BF*_ROPE_ and $P(ROPE|Data$) presented only for TAS-20 correlations. Bayes factors indicating substantial evidence against the interval null hypothesis (i.e., *r* or lies within [-0.2, 0.2]) are presented in bold, whereas Bayes factors indicating substantial evidence *for* the interval null hypothesis are presented in italics. Correlations are estimated using Bayesian methods and are presented along with 95% highest density credible intervals (CrI). *BF*_ROPE_ = Bayes factor assessing interval null hypothesis that the effect falls within the region of practical equivalence (ROPE); $P(ROPE|Data$) = proportion of the *r* posterior distribution falling within the ROPE, conditioned on the observed data (i.e., probability that the interval null hypothesis is true); SRS-2 = Social Responsiveness Scale–Second Edition; RBS-R = Repetitive Behavior Scale–Revised; SM = Sensory Motor (“lower order” repetitive behaviors) subscale; RS = Ritualistic/Sameness (“higher order” repetitive behaviors) subscale; BDI-II = Beck Depression Inventory–II; GAD-7 = Generalized Anxiety Disorder–7; BFNE-S = Brief Fear of Negative Evaluation–Short; PHQ-15 = (modified) Patient Health Questionnaire–15; WHOQOL-4 = Four Item World Health Organization Quality of Life Score; Suicidality = BDI-II item 9 (*Suicidal Thoughts or Wishes*); IPIP-N10 = ten-item neuroticism scale from the international personality item pool.

# **Supplemental References**

Beck, A. T., Steer, R. A., & Brown, G. K. (1996). *BDI-II, Beck Depression Inventory: Manual* (2nd ed). Psychological Corporation.

Bishop, S. L., Hus, V., Duncan, A., Huerta, M., Gotham, K., Pickles, A., Kreiger, A., Buja, A., Lund, S., & Lord, C. (2013). Subcategories of restricted and repetitive behaviors in children with autism spectrum disorders. *Journal of Autism and Developmental Disorders*, *43*(6), 1287–1297. https://doi.org/10.1007/s10803-012-1671-0

Bodfish, J. W., Symons, F. J., Parker, D. E., & Lewis, M. H. (2000). Varieties of repetitive behavior in autism: Comparisons to mental retardation. *Journal of Autism and Developmental Disorders*, *30*(3), 237–243. https://doi.org/10.1023/A:1005596502855

Boulton, K., & Guastella, A. (2020). Measuring social anxiety in adults with autism spectrum disorder: Psychometric properties of self-report instruments. *PsyArXiv*. https://doi.org/10.31234/osf.io/wakrb

Breen, R., Holm, A., & Karlson, K. B. (2014). Correlations and nonlinear probability models. *Sociological Methods & Research*, *43*(4), 571–605. https://doi.org/10.1177/0049124114544224

Brouwer, D., Meijer, R. R., & Zevalkink, J. (2013). On the factor structure of the Beck Depression Inventory-II: G is the key. *Psychological Assessment*, *25*(1), 136–145. https://doi.org/10.1037/a0029228

Bruni, T. P. (2014). Test Review: Social Responsiveness Scale–Second Edition (SRS-2). *Journal of Psychoeducational Assessment*, *32*(4), 365–369. https://doi.org/10.1177/0734282913517525

Bürkner, P.-C. (2017). brms: An R package for Bayesian multilevel models using Stan. *Journal of Statistical Software*, *80*(1), 1–28. https://doi.org/10.18637/jss.v080.i01

Capriola, N. N., Maddox, B. B., & White, S. W. (2017). No Offense Intended: Fear of Negative Evaluation in Adolescents and Adults with Autism Spectrum Disorder. *Journal of Autism and Developmental Disorders*, *47*(12), 3803–3813. https://doi.org/10.1007/s10803-016-2827-0

Carleton, R. N., Collimore, K. C., McCabe, R. E., & Antony, M. M. (2011). Addressing revisions to the Brief Fear of Negative Evaluation scale: Measuring fear of negative evaluation across anxiety and mood disorders. *Journal of Anxiety Disorders*, *25*(6), 822–828. https://doi.org/10.1016/j.janxdis.2011.04.002

Constantino, J. N., & Gruber, C. P. (2012). *Social Responsiveness Scale–Second Edition (SRS-2): Manual* (2nd ed.). Western Psychological Services.

Gelman, A., & Rubin, D. B. (1992). Inference from iterative simulation using multiple sequences. *Statistical Science*, *7*(4), 457–472. https://doi.org/10.1214/ss/1177011136

Goldberg, L. R., Johnson, J. A., Eber, H. W., Hogan, R., Ashton, M. C., Cloninger, C. R., & Gough, H. G. (2006). The international personality item pool and the future of public-domain personality measures. *Journal of Research in Personality*, *40*(1), 84–96. https://doi.org/10.1016/j.jrp.2005.08.007

Homan, M. D., & Gelman, A. (2014). The No-U-turn sampler: Adaptively setting path lengths in Hamiltonian Monte Carlo. *The Journal of Machine Learning Research*, *15*(1), 1593–1623. https://doi.org/10.5555/2627435.2638586

Hull, L., Mandy, W., Lai, M.-C., Baron-Cohen, S., Allison, C., Smith, P., & Petrides, K. V. (2019). Development and validation of the Camouflaging Autistic Traits Questionnaire (CAT-Q). *Journal of Autism and Developmental Disorders*, *49*(3), 819–833. https://doi.org/10.1007/s10803-018-3792-6

Kroenke, K., Spitzer, R. L., & Williams, J. B. W. (2002). The PHQ-15: Validity of a new measure for evaluating the severity of somatic symptoms. *Psychosomatic Medicine*, *64*(2), 258–266.

Kroenke, K., Spitzer, R. L., Williams, J. B. W., & Löwe, B. (2010). The Patient Health Questionnaire somatic, anxiety, and depressive symptom scales: A systematic review. *General Hospital Psychiatry*, *32*(4), 345–359. https://doi.org/10.1016/j.genhosppsych.2010.03.006

Kruschke, J. K. (2013). Bayesian estimation supersedes the t test. *Journal of Experimental Psychology: General*, *142*(2), 573–603. https://doi.org/10.1037/a0029146

Kurz, A. S. (2019, February 10). Bayesian robust correlations with brms (and why you should love Student’s t). *A. Solomon Kurz*. https://solomonkurz.netlify.app/post/bayesian-robust-correlations-with-brms-and-why-you-should-love-student-s-t/

Lam, K. S. L., & Aman, M. G. (2007). The Repetitive Behavior Scale-Revised: Independent validation in individuals with autism spectrum disorders. *Journal of Autism and Developmental Disorders*, *37*(5), 855–866. https://doi.org/10.1007/s10803-006-0213-z

Leary, M. R. (1983). A brief version of the Fear of Negative Evaluation Scale. *Personality and Social Psychology Bulletin*, *9*(3), 371–375. https://doi.org/10.1177/0146167283093007

Lewandowski, D., Kurowicka, D., & Joe, H. (2009). Generating random correlation matrices based on vines and extended onion method. *Journal of Multivariate Analysis*, *100*(9), 1989–2001. https://doi.org/10.1016/j.jmva.2009.04.008

Maddox, B. B., & White, S. W. (2015). Comorbid social anxiety disorder in adults with autism spectrum disorder. *Journal of Autism and Developmental Disorders*, *45*(12), 3949–3960. https://doi.org/10.1007/s10803-015-2531-5

McConachie, H., Mason, D., Parr, J. R., Garland, D., Wilson, C., & Rodgers, J. (2018). Enhancing the validity of a quality of life measure for autistic people. *Journal of Autism and Developmental Disorders*, *48*(5), 1596–1611. https://doi.org/10.1007/s10803-017-3402-z

McDermott, C. R., Farmer, C., Gotham, K. O., & Bal, V. H. (2020). Measurement of subcategories of repetitive behaviors in autistic adolescents and adults. *Autism in Adulthood*, *2*(1), 48–60. https://doi.org/10.1089/aut.2019.0056

Russell, A., Gaunt, D. M., Cooper, K., Barton, S., Horwood, J., Kessler, D., Metcalfe, C., Ensum, I., Ingham, B., Parr, J. R., Rai, D., & Wiles, N. (2020). The feasibility of low-intensity psychological therapy for depression co-occurring with autism in adults: The Autism Depression Trial (ADEPT) – a pilot randomised controlled trial. *Autism*, *24*(6), 1360–1372. https://doi.org/10.1177/1362361319889272

South, M., Carr, A. W., Stephenson, K. G., Maisel, M. E., & Cox, J. C. (2017). Symptom overlap on the SRS-2 adult self-report between adults with ASD and adults with high anxiety. *Autism Research*, *10*(7), 1215–1220. https://doi.org/10.1002/aur.1764

Spain, D., Happé, F., Johnston, P., Campbell, M., Sin, J., Daly, E., Ecker, C., Anson, M., Chaplin, E., Glaser, K., Mendez, A., Lovell, K., & Murphy, D. G. (2016). Social anxiety in adult males with autism spectrum disorders. *Research in Autism Spectrum Disorders*, *32*, 13–23. https://doi.org/10.1016/j.rasd.2016.08.002

Spitzer, R. L., Kroenke, K., Williams, J. B. W., & Löwe, B. (2006). A brief measure for assessing generalized anxiety disorder: The GAD-7. *Archives of Internal Medicine*, *166*(10), 1092–1097. https://doi.org/10.1001/archinte.166.10.1092

Tellegen, A., & Waller, N. G. (2008). Exploring personality through test construction: Development of the Multidimensional Personality Questionnaire. In G. J. Boyle, G. Matthews, & D. H. Saklofske (Eds.), *The SAGE Handbook of Personality Theory and Assessment: Personality Measurement and Testing* (Vol. 2, pp. 261–292). SAGE.

Watson, D., & Friend, R. (1969). Measurement of social-evaluative anxiety. *Journal of Consulting and Clinical Psychology*, *33*(4), 448–457. https://doi.org/10.1037/h0027806

Wetzels, R., & Wagenmakers, E.-J. (2012). A default Bayesian hypothesis test for correlations and partial correlations. *Psychonomic Bulletin & Review*, *19*(6), 1057–1064. https://doi.org/10.3758/s13423-012-0295-x

Williams, Z. J., Everaert, J., & Gotham, K. O. (2021). Measuring Depression in Autistic Adults: Psychometric Validation of the Beck Depression Inventory–II. *Assessment*, *28*(3), 858–876. https://doi.org/10.1177/1073191120952889

Williams, Z. J., & Gotham, K. O. (2021). Assessing general and autism‐relevant quality of life in autistic adults: A psychometric investigation using item response theory. *Autism Research*, 1–12. https://doi.org/10.1002/aur.2519

Witthöft, M., Fischer, S., Jasper, F., Rist, F., & Nater, U. M. (2016). Clarifying the latent structure and correlates of somatic symptom distress: A bifactor model approach. *Psychological Assessment*, *28*(1), 109–115. https://doi.org/10.1037/pas0000150
